# Anticancer Effects of Genistein and Its Interaction with Temozolomide in Human Melanoma, Neuroblastoma and Glioblastoma Cells

**DOI:** 10.3390/cimb48101030

**Published:** 2026-10-04

**Authors:** Paula Wróblewska-Łuczka, Bartłomiej Kwiatkowski, Maciej Kwieciński, Agnieszka Góralczyk, Robert Domitrović, Jarogniew J. Łuszczki

**Affiliations:** 1 Department of Occupational Medicine, Medical University of Lublin, ul. Jaczewskiego 8b, 20-090 Lublin, Poland; bartekchemlek@gmail.com (B.K.); m.kwiecinski.med@gmail.com (M.K.); agnieszka.goralczyk@umlub.edu.pl (A.G.); jarogniew.luszczki@umlub.edu.pl (J.J.Ł.); 2 Department of Medical Chemistry, Biochemistry and Clinical Chemistry, Faculty of Medicine, University of Rijeka, B. Branchetta 20, 51-000 Rijeka, Croatia; robert.domitrovic@uniri.hr

**Keywords:** genistein, temozolomide, isobolographic analysis

## Abstract

(1) Background: Melanoma, glioblastoma, and neuroblastoma have distinct cellular origins, biological characteristics, and clinical behavior. Although these tumors differ in their origins, they exhibit aggressive biological features and may develop treatment resistance. New therapeutic options are constantly being sought. This study aimed to evaluate the anticancer potential of genistein, a soy-derived isoflavone, and investigate the pharmacodynamic interactions between genistein and temozolomide in human melanoma, neuroblastoma, and glioblastoma cell lines. (2) Methods: Genistein’s potential for inhibiting cell viability, induction of cytotoxicity, and modulating apoptosis-related protein expression was assessed in eight cell lines. The interaction of the genistein + temozolomide combination (in a fixed 1:1 ratio) was assessed using isobolographic analysis. (3) Results: Genistein inhibited cell viability in a concentration-dependent manner. IC_50_ values ranged from approximately 40 to 110 μM in melanoma, 27 to 60 μM in neuroblastoma, and 37 to 83 μM in glioblastoma cell lines. Genistein treatment induced changes in apoptosis-related protein expression, including Bax and Bcl-2. The genistein + temozolomide combination produced additive interactions in the tested melanoma cell lines, whereas strong antagonism was observed in the neuroblastoma and glioblastoma models. These differences may reflect tumor-type-specific biological characteristics. (4) Conclusions: The findings provide comparative insights into genistein–temozolomide interactions across the investigated tumor models. Further studies are needed to clarify the underlying molecular mechanisms and evaluate the potential therapeutic relevance of this combination. Additional preclinical investigations are required to establish its efficacy, safety, and tolerability before clinical implications can be determined.

## 1. Introduction

Melanoma, glioblastoma, and neuroblastoma are malignant neoplasms with distinct cellular origins, biological characteristics, and clinical behavior [1,2,3,4]. Melanoma and neuroblastoma are associated with neural crest-derived cell populations, whereas glioblastoma originates from glial/neuroepithelial lineages of the neural tube and develops in the central nervous system. Neural crest cells migrate extensively during embryogenesis and give rise to diverse cell populations in anatomically distant regions of the developing organism [5,6]. These distinct developmental origins contribute to the biological heterogeneity of these tumors and may influence their molecular characteristics and responses to anticancer treatment. These tumors are characterized by aggressive biological behavior, substantial clinical challenges, and, depending on tumor type and disease context, invasive growth, metastatic dissemination, or resistance to anticancer treatment [7,8,9,10,11]. In this study, the term “neural-derived tumors” is used as a broad descriptive term referring to neuroblastoma and glioblastoma models, which represent tumors associated with the peripheral and central nervous systems, respectively, and do not share a common embryological origin.

In cancers for which complete surgical resection is not feasible, the use of radiotherapy or pharmacotherapy, often in combination with contemporary methods such as immunotherapy or targeted therapy, is imperative. However, standard chemotherapy remains a prevalent component of treatment in many cases [12]. It is of particular significance in the management of cancers that have advanced and are metastatic [12,13,14].

**Figure 1:**
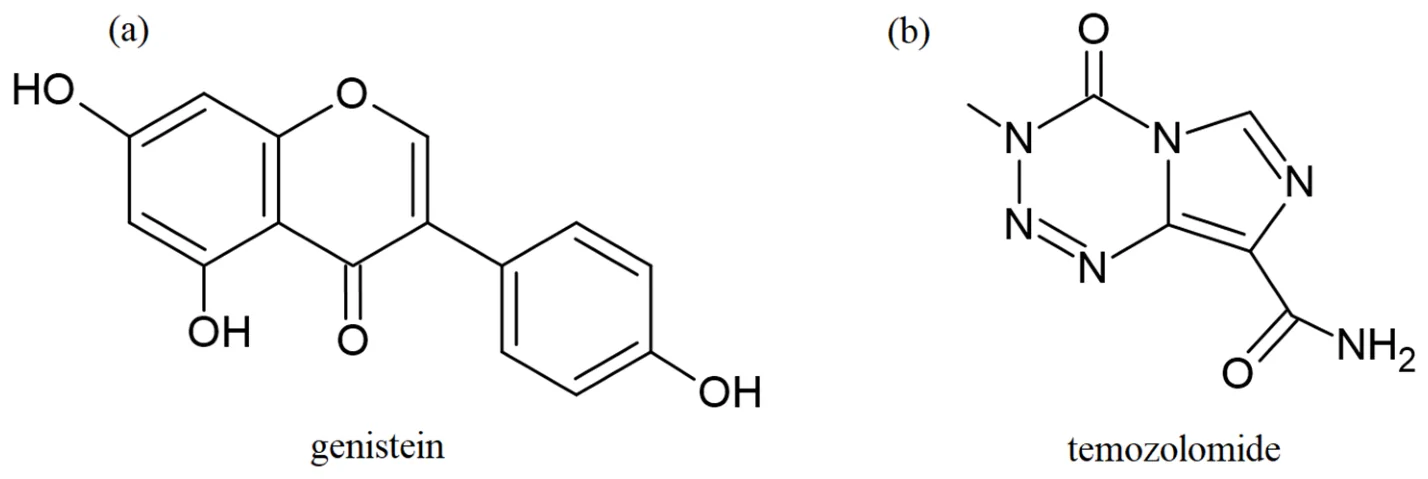
Chemical structure of (**a**) genistein and (**b**) temozolomide.

Glioma, glioblastoma and neuroblastoma primarily affect the central nervous system [8,9]. However, melanoma, a form of skin cancer, can metastasize to the brain in 10–40% of advanced cases [10,13]. This is particularly important when selecting appropriate, effective chemotherapeutic agents for the treatment of brain tumors, as they must be able to cross the blood–brain barrier (BBB) [15]. Temozolomide (TMZ) is currently used in the treatment of all of the aforementioned central nervous system tumors [7,13,14,16,17,18,19]. TMZ is a relatively novel alkylating agent (Figure 1b), a derivative of dacarbazine, which has been shown to cross the BBB and can also be administered orally [11,13,19]. Its effectiveness in cancer therapy is considered satisfactory, but like most cytostatic drugs, it can cause numerous side effects, including gastrointestinal disorders, neutropenia, thrombocytopenia, seizures, and neurological disorders [11,19,20].

In addition to standard surgical treatment, malignant tumors frequently necessitate aggressive and potentially harmful chemotherapy, and tumors may exhibit resistance to it. Consequently, there is a need to identify compounds that would serve to reduce the toxicity of chemotherapy while concomitantly increasing its effectiveness. It is noteworthy that over 60% of commonly used anticancer agents are derived from natural sources, including plants, marine organisms and microorganisms. Natural plant products play an active role in generating a significant number of medicinal compounds in the drug discovery programme [21,22]. Therefore, the exploration of novel therapeutic solutions derived from naturally occurring compounds appears to be a promising research direction, within which combinations of nutraceuticals with chemotherapeutics exhibiting synergistic and/or additive effects are increasingly being investigated.

Genistein (GEN) (4′,5,7-trihydroxyisoflavone—Figure 1a) is a flavonoid found primarily in soybeans, soybean sprouts, and other soy-derived products [23,24,25]. The present state of knowledge suggests that the benefits of GEN in the body include the inhibition of various processes, such as the formation of free radicals, the expression of inflammation, and the activity of tyrosine kinases. Other reported biological activities of genistein include the promotion of osteogenesis, inhibition of osteolysis, regulation of the cell cycle, and modulation of estrogen receptor signaling. These effects have been demonstrated in numerous in vitro and in vivo studies [24,25,26,27]. Consequently, genistein has been investigated for its potential therapeutic applications in a wide range of diseases and pathological conditions, including cardiovascular and neurodegenerative diseases, osteoporosis, cancer, polycystic ovary syndrome, and menopausal symptoms, as well as in promoting healthy aging [24,25,26,27,28]. Nevertheless, the most significant expectations are associated with the anti-cancer activity of GEN. Research on animal and human cell lines that would allow us to understand the mechanisms by which GEN inhibits carcinogenesis has been the subject of interest among scientists in recent years. Several mechanisms have been proposed to explain the anticancer activity of genistein. These include scavenging of free radicals and reactive oxygen species (ROS), activation of caspase-8, induction of mitochondria-mediated apoptosis, and inhibition of the nuclear factor kappa-light-chain-enhancer of activated B cells (NF-κB) and protein kinase B (Akt) signaling pathways [24,26,29,30,31,32,33]. Genistein has also been shown to downregulate the expression of BCL-2 at both the mRNA and protein levels while upregulating pro-apoptotic factors, including tumor necrosis factor receptor 1 (TNFR-1), Fas ligand (FasL), tumor necrosis factor alpha (TNF-α), and tumor necrosis factor receptor type 1-associated death domain protein (TRADD). In addition, genistein promotes lymphocyte proliferation and interferon-gamma (IFN-γ) release, increases p21 expression, decreases cyclin expression, and inhibits telomerase activity, ultimately leading to telomere shortening [24,26,29,30,31,32,33].

Unfortunately, due to low bioavailability, the use of this compound alone in cancer treatment would not be as effective as desired [33]. Therefore, combining GEN with clinically established cytostatic agents such as TMZ represents a promising strategy. This rationale is based on their distinct mechanisms of action, which may enhance the effectiveness of combination therapies. Genistein has been reported to modulate multiple cellular pathways relevant to temozolomide response, including apoptosis induction, oxidative stress regulation, and inhibition of pro-survival signaling pathways such as PI3K/AKT and MAPK. These properties suggest that genistein may influence the sensitivity of tumor cells to temozolomide, providing a rationale for investigating their combined effects.

Our study aimed to evaluate the anticancer potential of genistein in human melanoma and neural-derived tumor models, with particular emphasis on its effects on apoptosis. Furthermore, we investigated the pharmacodynamic interactions between GEN and TMZ in fixed 1:1 ratio combinations in melanoma and neural-derived tumor cell lines in vitro. To the best of our knowledge, this is the first study to directly compare genistein–temozolomide interactions across melanoma and neural-derived tumor models using isobolographic analysis. Understanding the nature of these interactions may provide valuable insights into the potential application of genistein as an adjuvant compound in anticancer therapy and may help to identify conditions under which combination treatment could enhance or reduce therapeutic efficacy.

## 2. Materials and Methods

### 2.1. Cell Lines

The study was conducted using human melanoma, glioblastoma, and neuroblastoma cell lines. Primary melanoma (FM55P) and metastatic melanoma (FM55M2), as well as two neuroblastoma cell lines (CHP-134 and KELLY), were obtained from the European Collection of Authenticated Cell Cultures (ECACC). Additionally, two melanoma cell lines—primary (A375) and metastatic (SK-MEL28)—and two glioblastoma cell lines (U-87MG and T98G) were purchased from the American Type Culture Collection (ATCC). In this study, HaCaT keratinocytes and primary rat astrocytes (normal healthy) [34]—as control cell lines—were used. Primary rat astrocytes were provided by Prof. Lemieszek from the Institute of Rural Health (Poland) and were obtained according to a previously published protocol [35]. The detailed information about the cell lines used in this research has been presented in the Supplementary Materials.

### 2.2. Drugs

Genistein (Sigma-Aldrich, St. Louis, MO, USA) was initially prepared as a 200 mM stock solution using dimethyl sulfoxide (DMSO) as the solvent. Temozolomide stock solutions were prepared in sterile water for injection at a concentration of 5 mg/mL. Prior to each experiment, both compounds were diluted in the appropriate culture medium to obtain the desired working concentrations. The final concentration of DMSO in the culture medium did not exceed 0.1%. Detailed information about these drugs is presented in the Supplementary Materials.

### 2.3. Cell Viability Assessment—MTT Assay

The impact of genistein and temozolomide, when administered alone and in combination, on cell viability was assessed in the MTT assay. Detailed information about this assay is presented in the Supplementary Materials and published earlier [34,36,37,38].

### 2.4. Cytotoxicity Assessment—LDH Assay

Genistein-induced cytotoxicity was determined by measuring lactate dehydrogenase (LDH) activity in the culture medium. The release of LDH from cells served as an indicator of membrane damage and loss of cell integrity. Detailed information about this assay is presented in the Supplementary Materials and described elsewhere [39,40].

### 2.5. Western Blot Analysis

Optimized amounts of primary melanoma (A375), metastatic melanoma (FM55M2) glioblastoma (T98G) and neuroblastoma (CHP-134) cell lines were placed in 6-well plates (Nest, Jiangsu, China). Twenty-four hours after seeding, the culture medium was aspirated and replaced with fresh medium containing genistein at concentrations corresponding to the IC_50_ values. Cells were incubated under these conditions for 72 h. Following treatment, cells were collected, rinsed twice with ice-cold phosphate-buffered saline (PBS), and lysed using RIPA buffer (Sigma-Aldrich, St. Louis, MO, USA) supplemented with a protease inhibitor cocktail (Sigma-Aldrich, St. Louis, MO, USA). The lysates were subsequently centrifuged at 14,000× *g* for 10 min at 4 °C. Protein concentrations were quantified using a BCA Protein Assay Kit (Thermo Scientific, Waltham, MA, USA) according to the manufacturer’s protocol. Equal amounts of protein were mixed with Laemmli sample buffer containing 5% β-mercaptoethanol and heated at 100 °C for 5 min. The prepared samples were then separated by SDS-PAGE and transferred onto polyvinylidene difluoride (PVDF) membranes (Bio-Rad Laboratories, Hercules, CA, USA) for further analysis. Next, the membranes were blocked in Tris-Buffered Saline (TBS) with 5% non-fat dry milk and 0.05% Tween 20, pH 7.5, for 2 h at room temperature (RT). Then, the membranes were incubated with the primary antibody: Bax and Bcl-2 (Cell Signaling Technology, Beverly, MA, USA) for 2 h at room temperature (RT). Primary antibodies were diluted in 5% (*w*/*v*) bovine serum albumin (BSA) according to the manufacturers’ recommendations. Following antibody incubation, membranes were washed twice with Tris-buffered saline containing 0.01% (*v*/*v*) Tween-20 (TBST) for 10 min each. Subsequently, membranes were incubated for 1 h at room temperature with the appropriate horseradish peroxidase (HRP)-conjugated secondary antibodies (Cell Signaling Technology, Beverly, MA, USA). After incubation, the membranes were washed twice again with TBST for 10 min per wash. Immunoreactive bands were detected using Pierce ECL Western Blotting Substrate (Thermo Scientific, Waltham, MA, USA) according to the manufacturer’s instructions and visualized with a Fusion FX imaging system (Vilber Lourmat, Marne-la-Vallée, France). Quantitative densitometric analysis was performed using FusionCapt Advance software. Protein expression levels were normalized to β-actin and presented as relative values. All experiments were conducted independently at least three times, and the results are expressed as mean ± standard error of the mean (SEM). Primary antibodies were used at a dilution of 1:1000, according to the manufacturer’s recommendations (Antibody from Cell Signaling (Danvers, MA, USA): beta-Actin Antibody #4967; Bax (E4U1V) Rabbit Monoclonal Antibody #41162; Bcl-2 (124) Mouse Monoclonal Antibody #15071; Cleaved Caspase-3 (Asp175) Rabbit Antibody #9661. Total cellular protein lysates were used for analysis without subcellular fractionation.

### 2.6. Isobolographic Analysis of Interactions

Pharmacodynamic interactions of genistein with temozolomide in tested cell lines were classified isobolographically in the MTT assay. Detailed information about the isobolographic analysis is presented in the Supplementary Materials (also on Supplementary Figure S1) and described elsewhere [37,41,42,43].

### 2.7. Statistical Analysis

Statistical analyses were performed using GraphPad Prism version 8.0 (GraphPad Software, San Diego, CA, USA). Differences between experimental groups were evaluated by one-way analysis of variance (ANOVA), followed by Dunnett’s post hoc test for multiple comparisons. Results are presented as mean ± standard error of the mean (SEM).

The half-maximal inhibitory concentrations (IC_50_) for genistein and temozolomide, as well as the corresponding IC_50mix_ values for their combinations at a fixed 1:1 ratio, were determined using computer-assisted log-probit analysis [41]. For each independent experiment, the eight technical replicates were first averaged at each concentration, and IC_50_ and IC_50mix_ were then estimated from the resulting experiment-level concentration–response data. To assess the nature of the interaction between the tested compounds, experimentally obtained IC_50mix_ values were compared with the theoretically calculated additive values (IC_50add_) using an unpaired Student *t*-test with Welch’s correction [42,43]. A detailed description of all statistical procedures applied in this study is provided in the Supplementary Materials.

### 2.8. Molecular Docking Analysis

Molecular docking studies were performed to investigate the predicted binding modes of genistein (GEN) and temozolomide (TMZ) to human O6-alkylguanine-DNA alkyltransferase (MGMT). The MGMT structure used for docking was obtained from the Protein Data Bank (PDB ID: 1EH6; chain A; resolution 2.0 Å) [44] and represented the native, unalkylated form of the catalytic residue Cys145. The alkylated conformation of MGMT was not modeled in the present study. Protein structure preparation was performed using UCSF ChimeraX version 1.12 [45]. Solvent molecules, non-complexed ions, and non-current alternate locations were removed, while hydrogen atoms were added using the steric-only hydrogen placement method. Partial atomic charges were assigned using the AM1-BCC method. The prepared receptor structure was subsequently saved as a PDB file and converted to PDBQT format using Meeko version 0.8.0 [46].

Genistein (PubChem CID 5280961) and temozolomide (PubChem CID 5394) were used as ligands. The molecular structures were obtained from PubChem in SDF format. TMZ was subjected to structure preparation in ChimeraX and subsequently converted to PDBQT format using Meeko. For GEN, the original PubChem SDF structure was directly converted to PDBQT format using Meeko. No salts or alternative protonation states of GEN were used.

Docking calculations were performed using AutoDock Vina version 1.2.7 [47,48]. The docking search space was defined as a cubic box of 20 × 20 × 20 Å centered at x = 3.0, y = 43.6, and z = 16.1 Å. The docking region was selected to encompass the area surrounding Cys145, the active-site nucleophilic residue of MGMT. The same docking box was used for both ligands to allow comparison of their predicted binding modes. The exhaustiveness parameter was set to 16, and up to 10 binding modes were generated for each ligand, with an energy range of 3 kcal/mol.

To assess the reproducibility of the docking results, docking calculations were performed in two independent runs for each ligand. The second run was performed using a fixed random seed (12,345). The top-ranked poses from both runs were subsequently analyzed in UCSF ChimeraX. Predicted ligand–protein contacts, potential hydrogen bonds, steric clashes, and distances between the ligands and the Cys145 sulfur atom were evaluated. The spatial relationship between the predicted GEN and TMZ binding poses was also examined.

## 3. Results

### 3.1. Effect of Genistein and Temozolomide on Cell Viability in the MTT Assay

**Figure 2:**
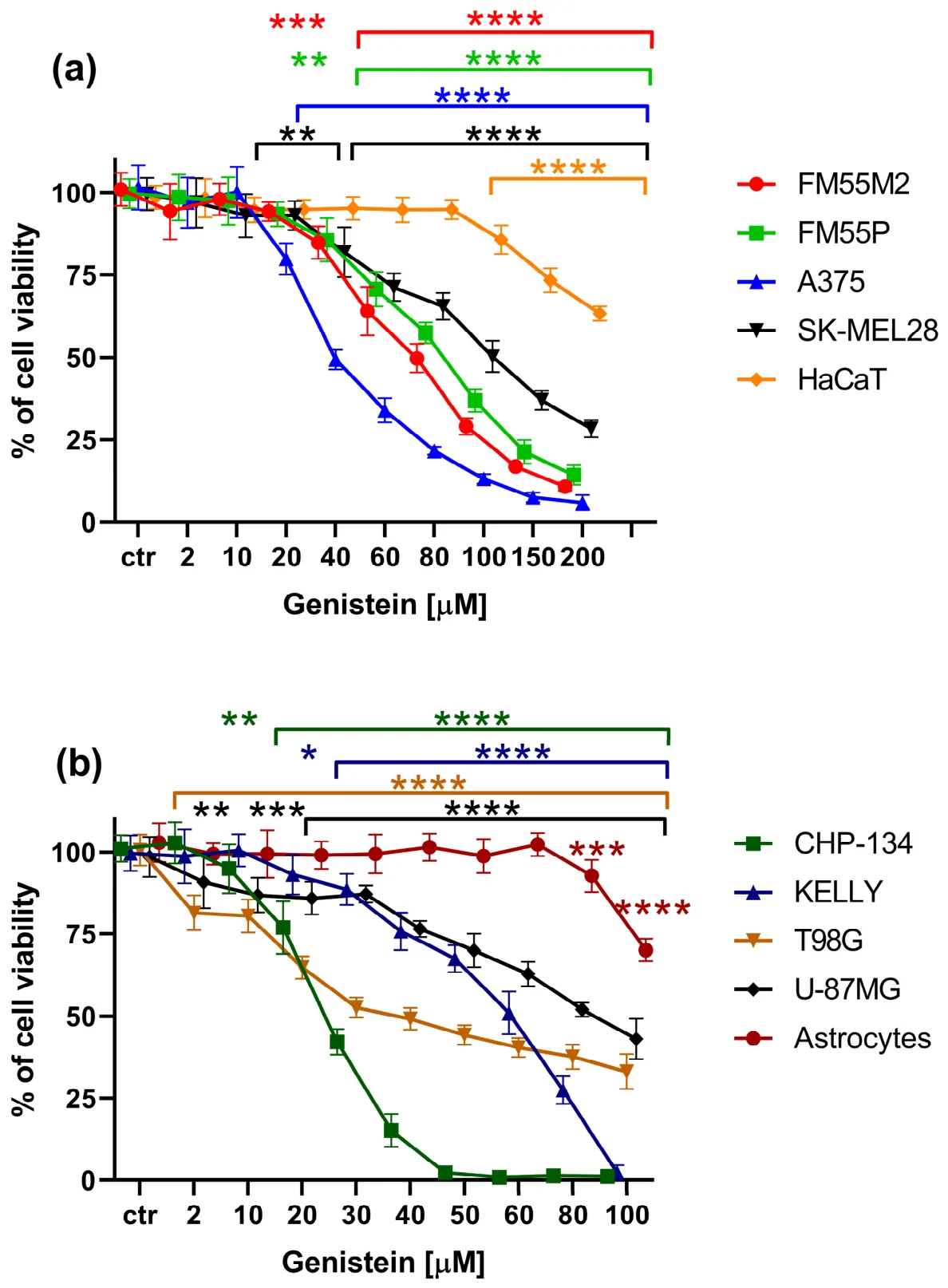
Concentration-dependent effects of genistein on the viability of (**a**) melanoma and HaCaT keratinocyte, (**b**) neuroblastoma, glioblastoma, and primary rat astrocyte, assessed by the MTT assay. Values are expressed as mean ± SEM from three independent experiments, each performed with eight technical replicates (24 total measurements per cell line). * *p* < 0.05, ** *p* < 0.01, *** *p* < 0.001, **** *p* < 0.0001 vs. the control (ctr) group (one-way ANOVA followed by Dunnett’s post-hoc test).

**Figure 3:**
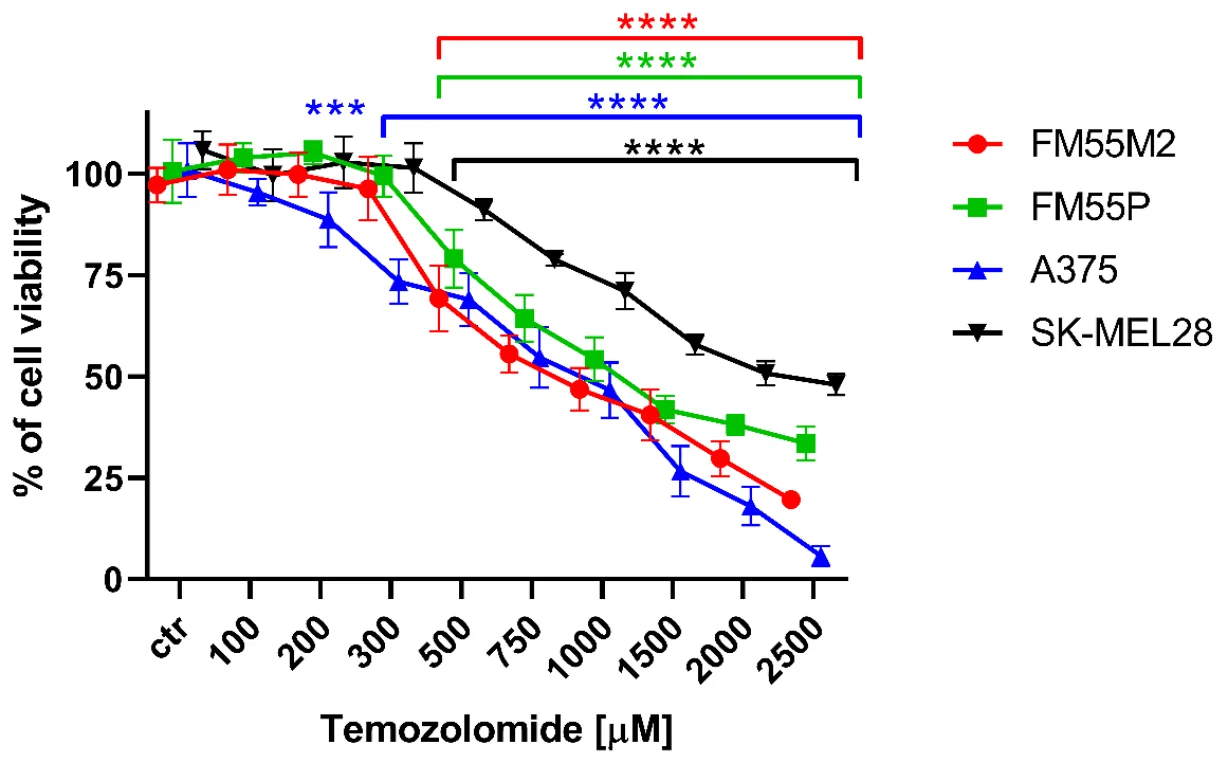
Concentration-dependent effects of temozolomide on the viability of melanoma cell lines, assessed by the MTT assay. Values are expressed as mean ± SEM from three independent experiments, each performed with eight technical replicates (24 total measurements per cell line). *** *p* < 0.001, **** *p* < 0.0001 vs. the control (ctr) group (one-way ANOVA followed by Dunnett’s post-hoc test).

Incubation of melanoma (FM55P, FM55M2, A375, SK-MEL28), neuroblastoma and glioblastoma cells (CHP-134, KELLY, U-87MGand T98G) with increasing concentrations of genistein resulted in the reduction in cell viability (Figure 2). Temozolomide also caused a dose-dependent inhibition of cell viability, as shown in the graphs for melanoma cells (Figure 3). The TMZ concentration–response relationship was independently re-evaluated in the present study for the neural-derived tumor cell lines. The newly obtained IC_50_ values were comparable to those previously reported [34] and were used to verify the concentrations applied in the subsequent combination experiments. The corresponding data are presented in Table 1 and Supplementary Figure S2.

In all melanoma cell lines tested, a statistically significant inhibition of cell viability was observed upon treatment with genistein from a concentration of 20 µM (Figure 2a), while in the case of neural-derived tumor cells from 2 µM in the case of glioblastoma (T98G, U-87MG) and 10–20 µM in the case of neuroblastoma (KELLY, CHP-134) (Figure 2b). In HaCaT keratinocytes, a statistically significant decrease in cell viability was observed at genistein concentrations of 100 µM and above (Figure 2a).

The experimentally determined median inhibitory concentrations (IC_50_ values) of the studied genistein and temozolomide in the all tested human melanoma, as well as the neuroblastoma and glioblastoma cell lines, are presented in Table 1. The lowest IC_50_ dose of genistein was observed for both the neuroblastoma CHP-134 (27.05 ± 1.78 µM) and glioblastoma T98G (37.63 ± 9.87 µM) cell lines. In melanoma cell lines, the lowest IC_50_ dose was observed for the primary A375 cell line (40.80 ± 5.09 µM), and the highest dose was observed for the metastatic melanoma SK-MEL28 cell line (110.09 ± 15.65 µM). For the normal HaCaT keratinocyte line and rat astrocytes, the IC_50_ values of genistein were estimated using computer-assisted log-probit regression analysis and were approximately 274 µM and 207 µM, respectively. Because 50% inhibition of cell viability was not reached within the tested concentration ranges, these values were extrapolated from the fitted concentration–response curves.

**Figure 4:**
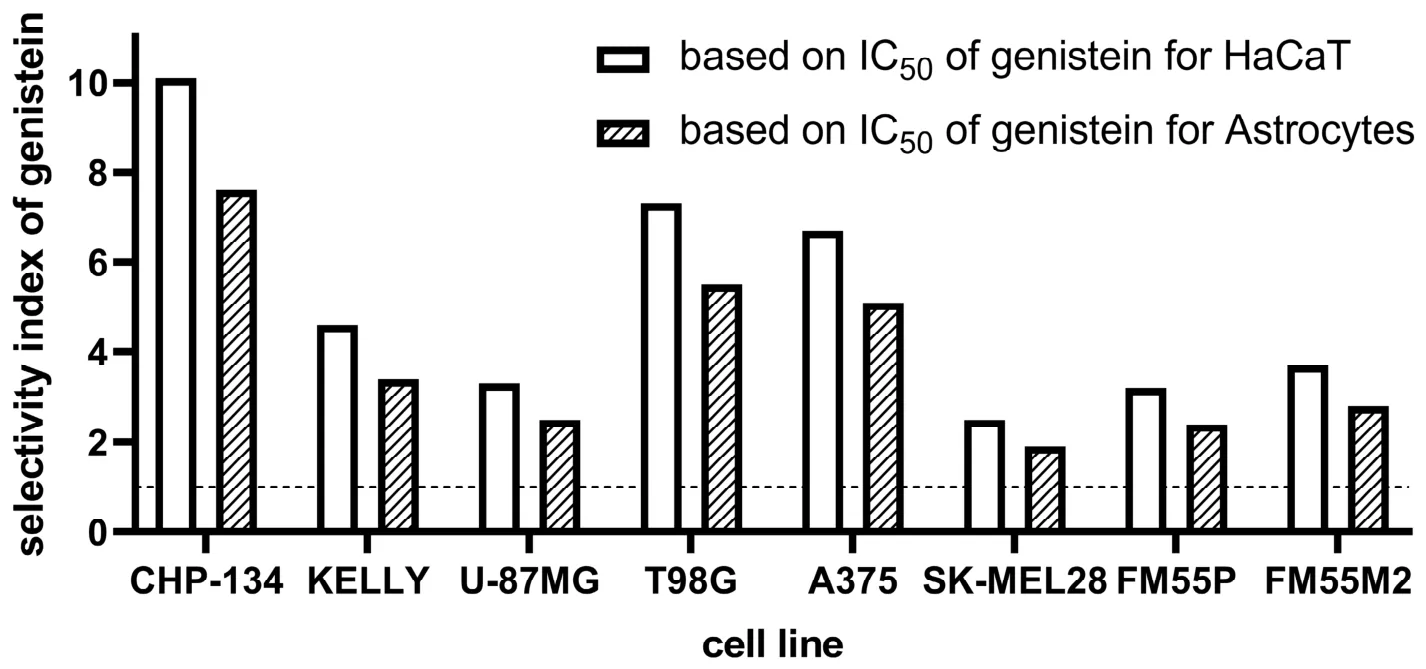
Selectivity index (SI) values for genistein determined in the MTT assay. The SI was calculated as the ratio of the IC_50_ value obtained for the normal cell line (HaCaT keratinocytes or primary rat astrocytes) to the corresponding IC_50_ value determined for each tested cancer cell line. The dashed line at SI = 1 represents the reference threshold for the selectivity index. SI values >1 indicate greater selectivity toward tumor cells than toward normal cells.

The selectivity index (SI) was calculated to assess the relative selectivity of genistein toward cancer cells compared with normal cells. SI values were determined by dividing the IC_50_ obtained for HaCaT keratinocytes or primary rat astrocytes by the corresponding IC_50_ values for each tested cancer cell line. SI values greater than 1 indicate that a higher concentration of GEN was required to reduce metabolic activity by 50% in the comparator normal cells than in the corresponding cancer cells. Therefore, the obtained results suggest a degree of preferential cytotoxic activity of GEN toward the tested cancer cell lines under the experimental conditions used. The SI was above 3 for the two neuroblastoma cell lines, above 2 for the two glioblastoma cell lines, and above 2 for three melanoma cell lines. For the SK-MEL28 cell line, the SI calculated using the IC_50_ of GEN in primary rat astrocytes was 1.9 (Figure 4). However, these SI values should be interpreted as indicators of relative in vitro selectivity and not as evidence of the overall safety or non-toxicity of GEN. The safety and clinical tolerability of GEN require further evaluation in appropriate in vivo, pharmacokinetic, and clinical studies.

### 3.2. Cytotoxicity of Genistein in the LDH Test

The cytotoxicity of genistein to tested melanoma, neuroblastoma and glioblastoma cell lines was measured by LDH assay. The LDH assay was used to evaluate lactate dehydrogenase release into the culture medium, which serves as a marker of cell membrane damage and cell death [49].

**Figure 5:**
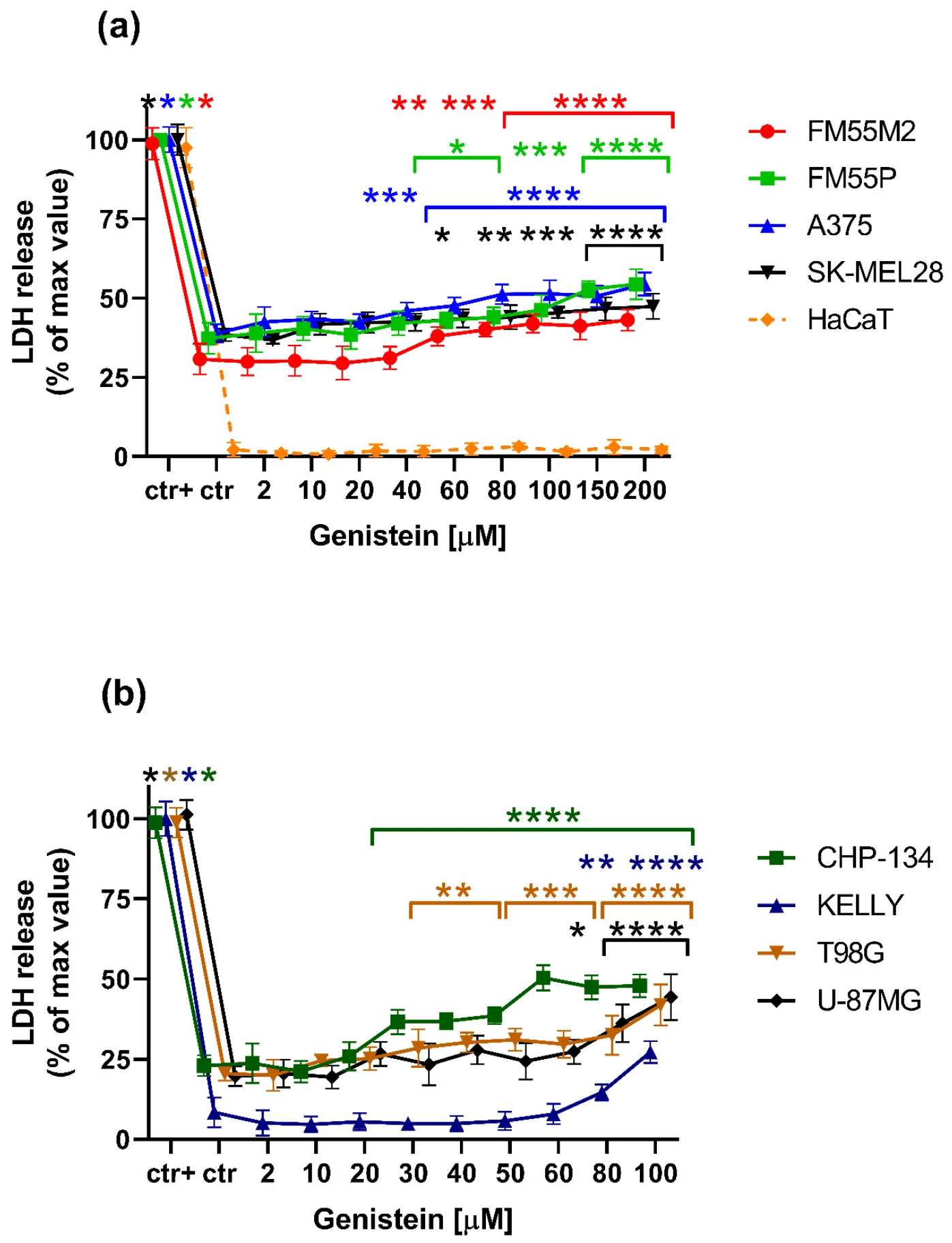
Impact of genistein on cytotoxicity in melanoma and HaCaT keratinocytes (**a**), as well as neuroblastoma and glioblastoma (**b**) cell lines, in the LDH assay. Columns represent the means ± SEMs. Each group was tested in three independent experiments, each performed in eight technical replicates (24 total measurements per cell line). * *p* < 0.05, ** *p* < 0.01, *** *p* < 0.001, and **** *p* < 0.0001 vs. the control (ctr) group; ctr+—cells treated with lysis buffer (one-way ANOVA followed by Dunnett’s post-hoc test).

In the present study, treatment with genistein resulted in a significant increase in LDH release in melanoma cell lines at concentrations ranging from 40–60 μM up to 200 μM (Figure 5a). In the case of neural-derived tumors, statistically significant LDH leakage was observed from genistein concentrations of 30–40 μM for the cell lines with the lowest IC_50_ doses, i.e., neuroblastoma CHP-134 and glioblastoma T98G. In the case of the other two neurological tumor cell lines, U-87MG and KELLY, statistically significant LDH leakage was observed from concentrations of 60 μM and 80 μM, respectively (Figure 5b). However, these results should be interpreted cautiously, as the relatively high spontaneous LDH release observed in some untreated controls reduced the dynamic range of the assay.

### 3.3. Western Blotting Analysis

**Figure 6:**
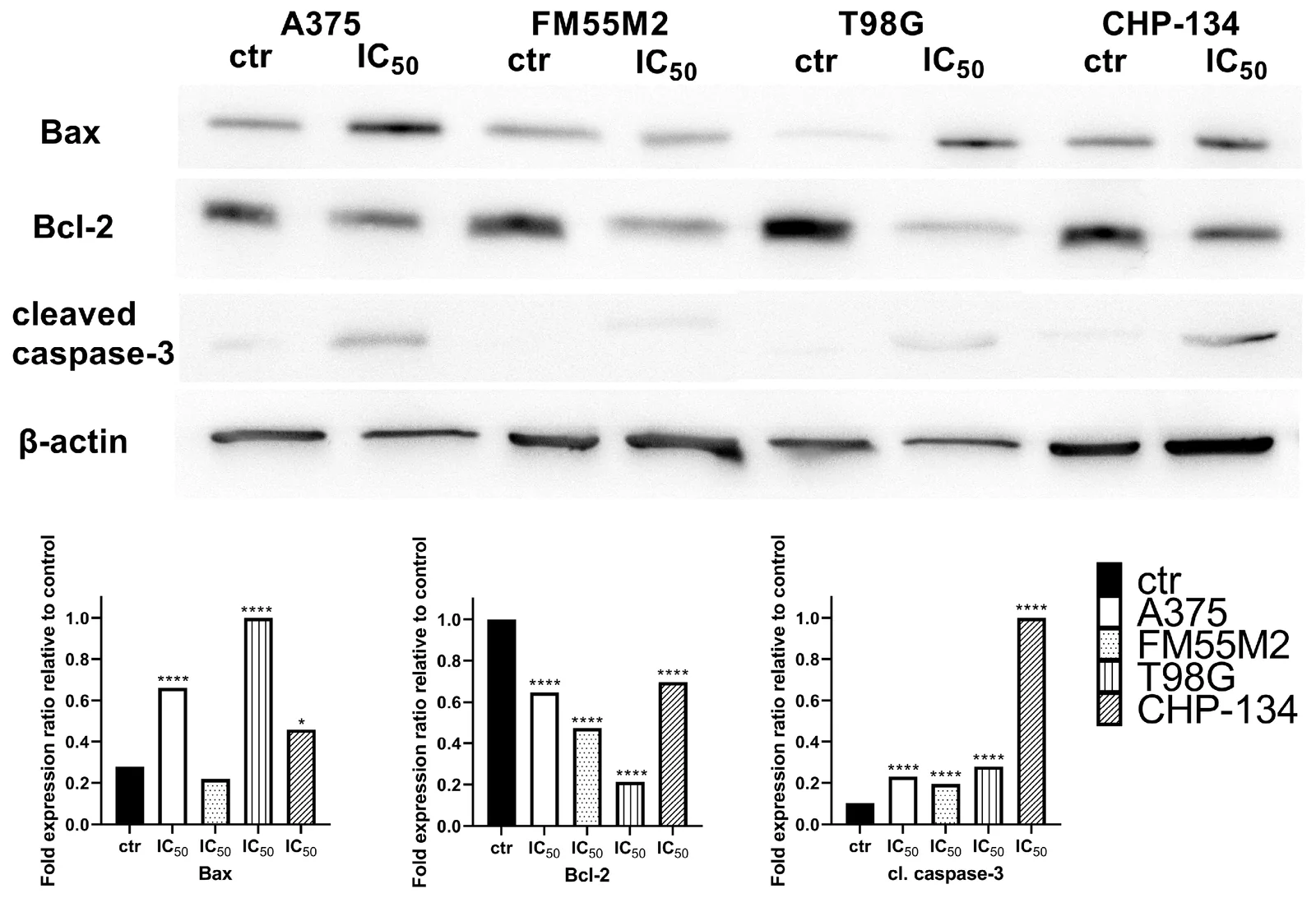
Representative blots of Bax, Bcl-2 and cleaved caspase-3 proteins following incubation of melanoma (A375, FM55M2), glioblastoma (T98G) and neuroblastoma (CHP-134) cells with either culture medium alone (ctr) or genistein at IC_50_ concentration. β-actin was used as a gel loading control. Densitometric analysis of the bands was performed by FusionCapt Advance softwere. * *p* < 0.05, **** *p* < 0.0001 vs. the control (ctr) group (one-way ANOVA followed by Dunnett’s post-hoc test).

To investigate the molecular mechanisms potentially involved in the cellular response to genistein treatment, apoptosis-related proteins were examined. Four cell lines with the lowest IC_50_ values for genistein were selected for the experiments, including two melanoma cell lines—one primary (A375) and one metastatic (FM55M2)—one glioblastoma cell line (T98G), and one neuroblastoma cell line (CHP-134). Western blot analysis revealed cell line-dependent changes in the expression of the apoptosis-related proteins Bcl-2 and Bax following treatment with genistein at the IC_50_ concentration. Although Bax expression increased in some cell lines, a decrease in Bax protein levels was observed in FM55M2 cells. Changes in the relative expression of Bax and Bcl-2 resulted in an increased Bax/Bcl-2 ratio in all tested cell lines. Moreover, genistein treatment significantly increased the expression of cleaved caspase-3 in all tested cell lines, supporting the activation of apoptosis-related signaling following genistein treatment. Densitometric analysis revealed a 2.3-fold increase in A375 cells, a 1.9-fold increase in FM55M2 cells, a 2.8-fold increase in T98G cells, and a 10-fold increase in CHP-134 cells compared with the untreated control (all *p* < 0.0001), further supporting the involvement of apoptosis-related signaling in the cellular response to genistein treatment (Figure 6). The original, uncropped Western blot images, including the molecular-weight markers and loading controls, as well as the corresponding densitometric analysis results, are provided in the Supplementary Materials (Supplementary Figures S3–S5 and Supplementary Table S1).

### 3.4. Isobolographic Interactions Between Genistein and Temozolomide in the MTT Assay

The test of the parallelism of log-probit concentration–response inhibitory effects of genistein and temozolomide revealed that genistein had its log-probit concentration–response inhibitory effect line collateral to that of temozolomide in four neurological cancers: glioblastoma (U-87MG and T98G), neuroblastoma (CHP-134, KELLY) (Supplementary Figure S6) and two melanoma cell lines (A375, SK-MEL28) (Supplementary Figure S7). Genistein had its log-probit concentration–response inhibitory effect line non-parallel only in two melanoma cell lines: FM55P and FM55M2 (Supplementary Figure S7). The MTT concentration–response data for the GEN+TMZ mixture at a fixed 1:1 ratio are shown in Supplementary Figures S8 and S9. These data were used for computer-assisted log-probit regression analysis and determination of the experimentally derived IC_50mix_ values.

**Figure 7:**
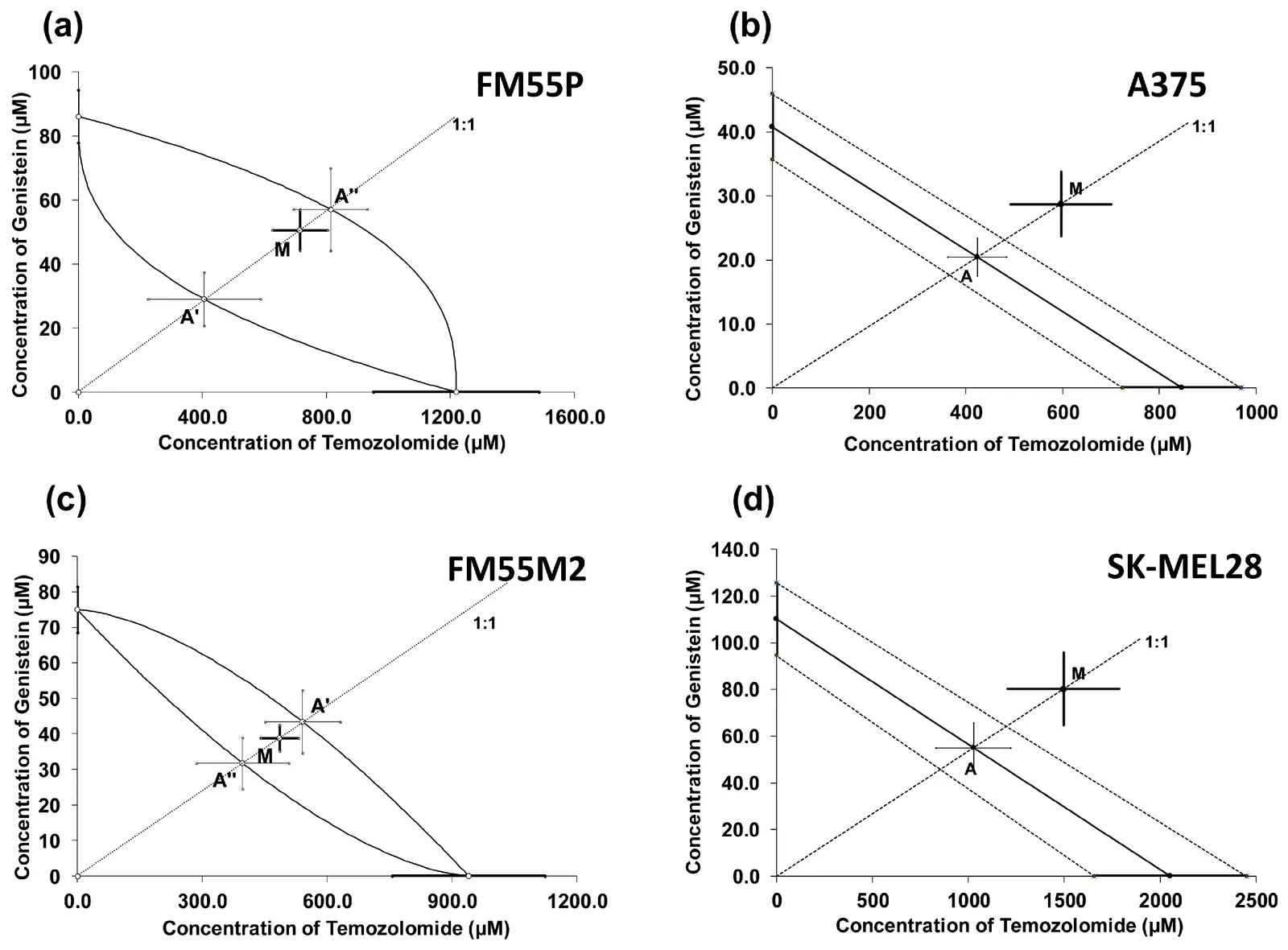
Isobolographic analysis of the interaction between genistein and temozolomide in FM55P (**a**), A375 (**b**), FM55M2 (**c**), and SK-MEL-28 (**d**) melanoma cell lines, based on MTT assay results. IC_50_ values (±SEM) for both compounds administered individually were plotted on Cartesian coordinates. Theoretical additive IC_50_ values (IC_50add_) are represented by points A (parallel concentration–response curves) or A′ and A″ (non-parallel concentration–response curves), whereas experimentally determined IC_50mix_ values for the drug combination are indicated by point M. Statistical comparisons between IC_50mix_ and IC_50add_ values were performed using an unpaired Student *t*-test with Welch’s correction. Figure 7b,d: The dashed lines represent the corresponding 95% confidence intervals, the area between the dashed lines indicates the additive interaction region.

The interaction between genistein and temozolomide, evaluated by isobolographic analysis at a fixed 1:1 concentration ratio, was classified as additive in all tested melanoma cell lines (Figure 7a–d). Nevertheless, in A375 and SK-MEL-28 cells, the experimentally obtained results were positioned close to the boundary of antagonism, indicating a slight trend toward an antagonistic interaction (Figure 7b,d).

**Figure 8:**
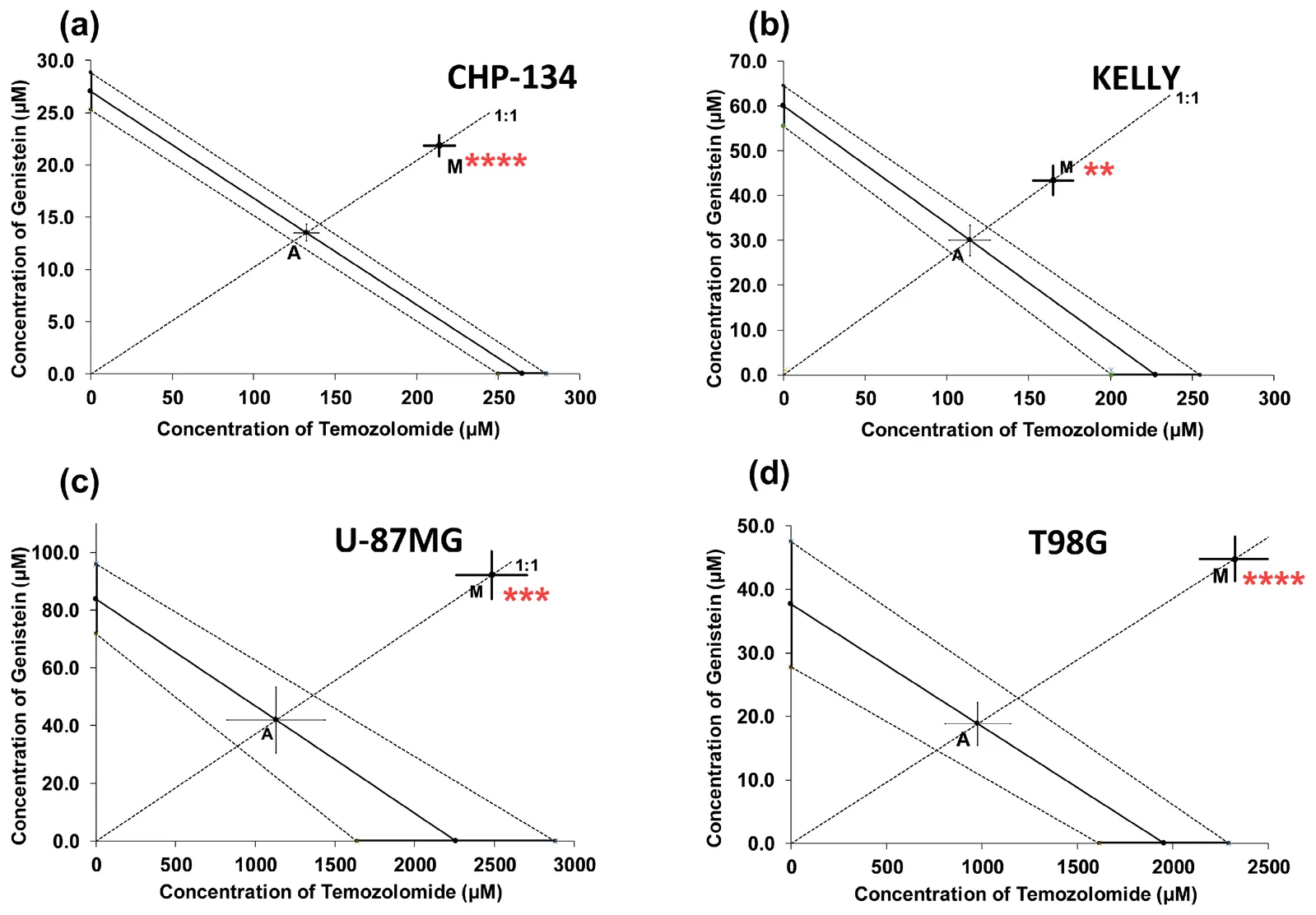
Isobolographic analysis of the interaction between genistein and temozolomide in CHP-134 (**a**), KELLY (**b**) neuroblastoma, and U-87MG (**c**) and T98G (**d**) glioblastoma cell lines, based on MTT assay results. IC_50_ values (± SEM) for both compounds administered individually were plotted on Cartesian coordinates. Theoretical additive IC_50_ values (IC_50add_) are represented by points A (parallel concentration–response curves) or A′ and A″ (non-parallel concentration–response curves), whereas experimentally determined IC_50mix_ values for the drug combination are indicated by point M. Statistical comparisons between IC_50mix_ and IC_50add_ values were performed using an unpaired Student *t*-test with Welch’s correction. (** *p* < 0.01, *** *p* < 0.001 and **** *p* < 0.0001). The dashed lines represent the corresponding 95% confidence intervals, the area between the dashed lines indicates the additive interaction region.

Moreover, the combination of genistein with temozolomide (at a fixed ratio of 1:1) exerted antagonistic interaction in four neurological cancers: neuroblastoma: CHP-134, *p* < 0.0001 (Figure 8a), KELLY, *p* < 0.01 (Figure 8b) and glioblastoma: U-87MG, *p* < 0.001 (Figure 8c) and T98G, *p* < 0.0001 (Figure 8d).

### 3.5. Molecular Docking Analysis of GEN and TMZ with MGMT

**Figure 9:**
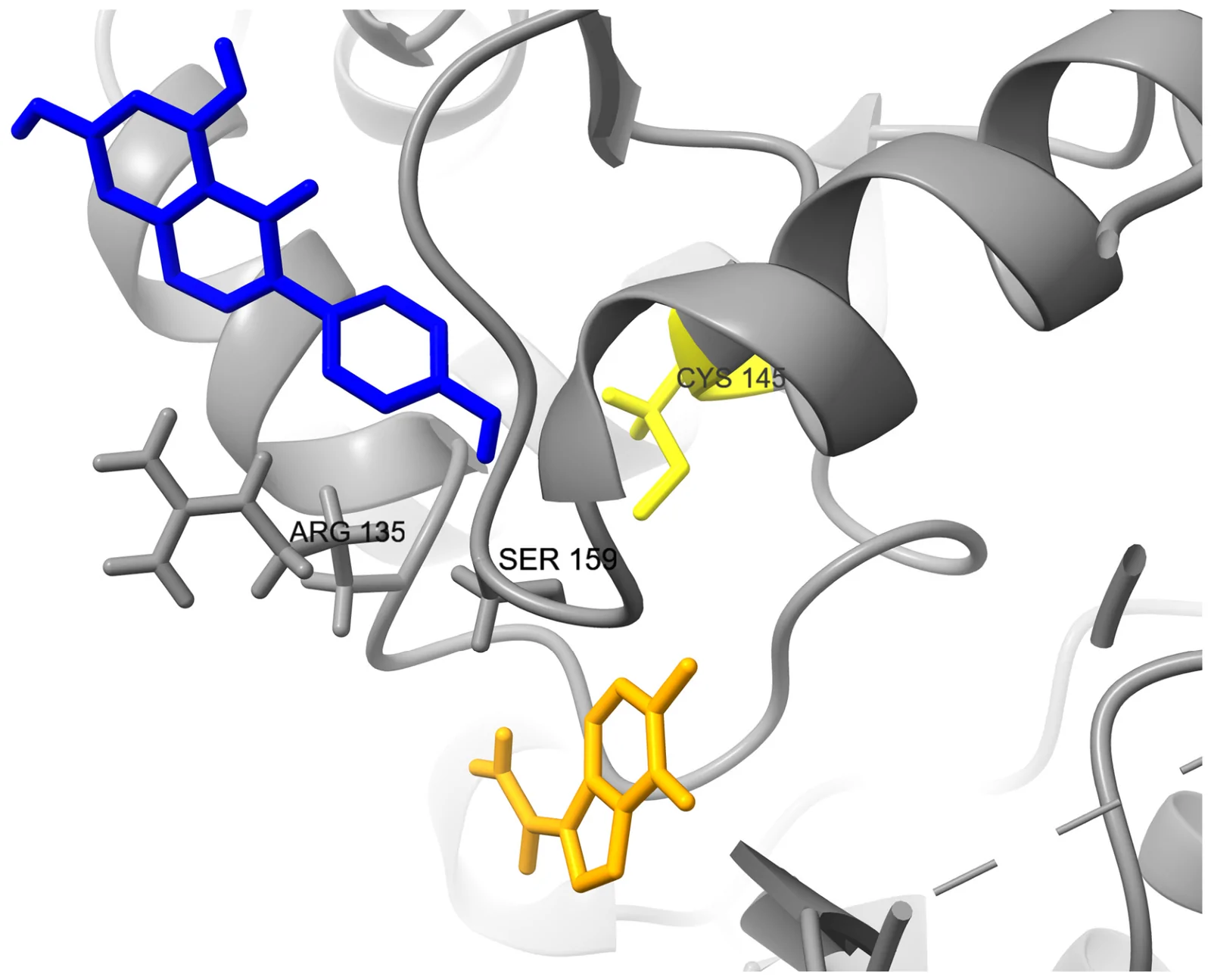
Molecular docking of genistein and temozolomide to human O6-alkylguanine-DNA alkyltransferase (MGMT). Predicted binding poses of genistein (GEN, blue) and temozolomide (TMZ, orange) within the broader MGMT region surrounding the active-site Cys145. Selected MGMT residues identified among the contacting residues, including Arg135 and Ser159, are shown as sticks. Cys145 is highlighted in yellow. Neither GEN nor TMZ showed a direct contact with Cys145 in the top-ranked docking poses.

Molecular docking analysis revealed reproducible binding poses for both genistein (GEN) and temozolomide (TMZ) within a broader region of the MGMT structure surrounding Cys145 (Figure 9). The docking calculations were performed using the native, unalkylated form of Cys145 in the MGMT structure deposited under PDB ID 1EH6. The top-ranked pose of GEN showed a predicted binding energy of −5.79 kcal/mol, whereas the corresponding value for TMZ was −5.50 kcal/mol. In independent docking runs performed using the same parameters, predicted binding energies were −5.79 and −5.79 kcal/mol for GEN and −5.50 and −5.50 kcal/mol for TMZ, respectively, indicating the good reproducibility of the predicted binding modes. The small difference in predicted binding energies between GEN and TMZ should not be interpreted as evidence of different binding affinities.

Analysis of the predicted binding poses showed that neither GEN nor TMZ formed a direct contact with Cys145. The minimum distance between the sulfur atom of Cys145 and the ligand was approximately 4.9–5.1 Å for GEN and 5.47 Å for TMZ. These distances do not support a direct, close interaction with the catalytic cysteine and therefore do not provide evidence of direct competitive inhibition of the MGMT active site. Both ligands were located within the same broader region of the MGMT structure, although they occupied adjacent subregions. Arg135 and Ser159 were identified among the residues surrounding both docked ligands. The top-ranked GEN and TMZ poses did not exhibit direct ligand–ligand contacts, with the minimum distance between the two ligands being approximately 5.9–6.0 Å. A summary of the main docking parameters is provided in Supplementary Table S2.

Potential hydrogen-bond interactions were also analyzed. For TMZ, two potential hydrogen bonds involving MGMT residues Leu33 and Ser159 were identified in both independent docking runs, supporting the reproducibility of these predicted interactions. In contrast, one potential hydrogen bond between GEN and Ser159 was identified in the first docking run but was not reproduced in the second run. Thus, the predicted interaction of GEN with Ser159 should be considered less robust than the corresponding interactions predicted for TMZ. These predicted hydrogen bonds should be interpreted as possible non-covalent interactions rather than experimental evidence of direct ligand binding or MGMT inhibition.

It should also be noted that intact TMZ was used as the docking ligand. Since TMZ undergoes chemical conversion in cells to generate reactive methylating species, docking of the parent TMZ molecule does not reproduce the covalent alkylation reaction of Cys145 or the subsequent conformational changes associated with MGMT inactivation. Therefore, the present docking results should be considered exploratory and hypothesis-generating, rather than evidence of direct inhibition of MGMT by TMZ or GEN.

## 4. Discussion

### 4.1. Mechanisms of Action of Genistein

GEN is a plant-derived compound belonging to the isoflavone group. This substance occurs naturally in significant quantities in legumes, particularly soybeans. Its chemical structure is analogous to oestrogen, thus classifying it as a phytoestrogenic compound. The potential for interaction with the oestrogen receptor has led to its consideration as a possible treatment for symptoms of menopause, osteoporosis and polycystic ovary syndrome (PCOS) [50]. GEN has undergone extensive research in various cancer treatments, including: breast, prostate, ovarian, cervical, colon, and lung cancers, as well as in hepatocellular carcinoma and leukaemia [51].

Despite the efficacy of available treatment options, cancer remains a serious condition due to the presence of cancer stem cells that are resistant to standard treatment. GEN belongs to the group of five compounds known as the ‘big five’ (the others being curcumin, epigallocatechin-3-gallate, sulforaphane and resveratrol), which have been shown to be capable of eliminating cancer stem cells [52].

GEN has been shown to exhibit anticancer activity through multiple mechanisms of action. One of the mechanisms frequently associated with the anticancer activity of GEN is the activation of apoptosis-related signaling, which may occur through several pathways. The DNA-PKcs/Akt2/Rac1 pathway has been demonstrated to be associated with the repair and migration of cancer cells. Overactivity of this pathway has been observed in a variety of conditions, including gliomas, and is associated with increased resistance to radio- and chemotherapy. GEN has been reported to selectively target DNA-PKcs, resulting in the downregulation of the DNA-PKcs/Akt2/Rac1 pathway. Consequently, treatment with genistein effectively attenuated the invasive and migratory properties of glioblastoma multiforme cells following irradiation in both in vitro and in vivo experimental settings [53]. The action of GEN has been shown to be associated with the activation of calpain 1, which in turn promotes the cleavage of the Bcl-2-associated protein X (Bax) and the BH3 domain death agonist (Bid). Activation of this pathway promotes the translocation of Bax and truncated Bid (t-Bid) to the mitochondrial membrane, triggering cytochrome c release into the cytoplasm and subsequent apoptosome assembly [54]. In addition, genistein has been reported to induce apoptosis through the inhibition of aerobic glycolysis by reducing the expression of HIF-1α, GLUT1, and hexokinase 2 (HK2). Furthermore, it has been indicated that the aforementioned process promotes phospho-ATM/phospho-ATR/p53/p21 signalling events, which are involved in the induction of apoptosis and cell cycle arrest processes [54]. MMP-2 and MMP-9 are among the most significant matrix metalloproteinases in the context of glioblastoma multiforme. The primary function of these proteins is the degradation of the extracellular matrix (ECM), a process that promotes the invasion and migration of tumor cells, thereby enhancing their metastatic potential. GEN has been revealed to inhibit the process of extracellular matrix degradation by interacting with the catalytic domain of MMP2 and MMP9, thereby limiting the invasive process of glioblastoma cells [55,56]. In a single study, GEN enhanced the action of TNF-related apoptosis-inducing ligand (TRAIL), facilitating TRAIL-dependent apoptosis at the level of extrinsic apoptotic pathways in malignant glioma cells [57]. Additionally, GEN has been shown to inhibit the expression of vascular endothelial growth factor (VEGF) in mesenchymal stem cells derived from both high-grade and low-grade gliomas, suggesting its potential to impair angiogenesis during tumor progression [58]. In a particular study, GEN was found to be capable of arresting the cell cycle at the G2/M phase, inhibiting telomerase, consequently leading to telomere shortening in glioblastoma cells [31].

In addition to the aforementioned mechanisms, further cellular processes have also been observed in melanoma cells. The analysis proved that GEN at concentrations ranging from 12.5 to 100 μM, exhibited a diminished expression of FAK, paxillin, vimentin, and Snail transcription factor genes. These genes are associated with the epithelial–mesenchymal transition (EMT) process in melanoma cells. A substantial decrease in the concentration of phosphorylated proteins linked to the MAPK p-p38, p-ERK and p-JNK pathways was evident. It is important to note that this effect was only achieved at high concentrations; at low concentrations, the opposite effect was observed—activation of the MAPK and FAK/paxillin pathways [59]. GEN has also been demonstrated to exhibit immunomodulatory properties. Prostaglandin E2 (PGE2) induces IL-8 synthesis via activation of the EP3 receptor; however, GEN has been reported to downregulate the expression of this receptor, along with mRNA expression and IL-8 levels. Consequently, it has been shown to inhibit melanoma cell proliferation [60]. GEN has been shown to impede tyrosine phosphorylation by suppressing tyrosinase activity in melanoma cells, a process of paramount importance given that tyrosine serves as a precursor for melanin [61]. In a melanoma-bearing mouse model, Farina et al. reported that treatment with GEN at a dose of 10 mg/kg/day markedly suppressed tumor-induced angiogenesis. Notably, a similar reduction in angiogenic activity was observed in mice fed a soy-based diet, indicating that genistein may interfere with processes involved in melanoma progression and metastasis [62]. GEN’s characteristic chemical structure affords it the ability to act as an antioxidant, thereby offering a protective effect against UV rays. Its mode of action is analogous to that of superoxide dismutase, which involves the elimination of oxygen free radicals [63].

Another experiment revealed that GEN exerts an apoptotic effect on prostate cancer cell lines PC-3 and LNCaP by upregulating p21 expression and (consequently) inhibiting PLK-1, which promotes apoptosis of cancer cells [64]. An investigation conducted on hepatocellular carcinoma (Hep3B) indicates that GEN induced a proapoptotic effect caused by endoplasmic reticulum stress, resulting in an increase in the concentrations of both caspase 2 and caspase 12. The anticancer activity of GEN was found to be associated with an increase in the production of reactive oxygen species (ROS) [65].

In the present study, the increased Bax/Bcl-2 ratio and elevated levels of cleaved caspase-3 following genistein treatment support the activation of apoptosis-related signaling. However, these findings alone do not establish apoptosis as the definitive mode of cell death. Previous studies have shown that genistein may activate different forms of regulated cell death depending on the cellular context. In particular, genistein has been reported to induce ferroptosis through the FoxO3/SLC7A11/GPX4 signaling pathway in colorectal cancer cells and to promote ferroptosis in MDA-MB-231 breast cancer cells [66,67]. Other studies have demonstrated that genistein may induce both apoptosis and autophagy in breast and ovarian cancer cells [68,69]. Therefore, although our findings support the activation of apoptosis-related signaling in the investigated melanoma, glioblastoma, and neuroblastoma cells, they do not exclude the involvement of other cell-death pathways. Further studies using pathway-specific markers and functional assays are required to determine the contribution of apoptosis, ferroptosis, autophagy, or other regulated cell-death mechanisms in these models.

Our experiments further demonstrate the concentration-dependent inhibitory effects of GEN on the viability of melanoma and neural-derived tumor cells. GEN inhibited the viability of the tested cell lines in a concentration-dependent manner; in the case of melanomas, the IC_50_ of genistein ranged from approximately 40 μM (the primary melanoma cell line A375) to approximately 110 μM (the metastatic human melanoma cell line SK-MEL28). Neural-derived tumors appear to be more sensitive to GEN, and the IC_50_ of GEN in neuroblastoma ranged from approximately 27 to 60 μM (for the two lines CHP-134 and KELLY, respectively), while in glioblastoma the IC_50_ of GEN ranged from approximately 37 to 83 μM (T98G and U-87MG cell lines, respectively). GEN demonstrated concentration-dependent cytotoxicity against all tested cancer cell lines, with IC_50_ values ranging from approximately 27 to 110 μM depending on the tumor type. Normal HaCaT keratinocyte line showed lower susceptibility to genistein-induced cytotoxicity. Although GEN exhibited significant anticancer activity in vitro, the IC_50_ values observed in the present study were relatively high compared with plasma concentrations typically achieved following dietary soy intake. Thus, the observed effects cannot be directly extrapolated to conventional soy consumption or standard genistein supplementation. Further in vivo and pharmacokinetic studies are required to establish the translational relevance of these findings. Pharmacokinetic studies have demonstrated that circulating concentrations of free GEN are generally considerably lower than those used in most in vitro experiments. Therefore, the translational potential of GEN may depend, at least in part, on the development of optimized delivery strategies, such as nanoformulations, targeted delivery systems, or local administration approaches, that could increase tumor exposure to GEN. However, the efficacy, safety, and pharmacokinetic feasibility of such approaches require further experimental validation.

**Figure 10:**
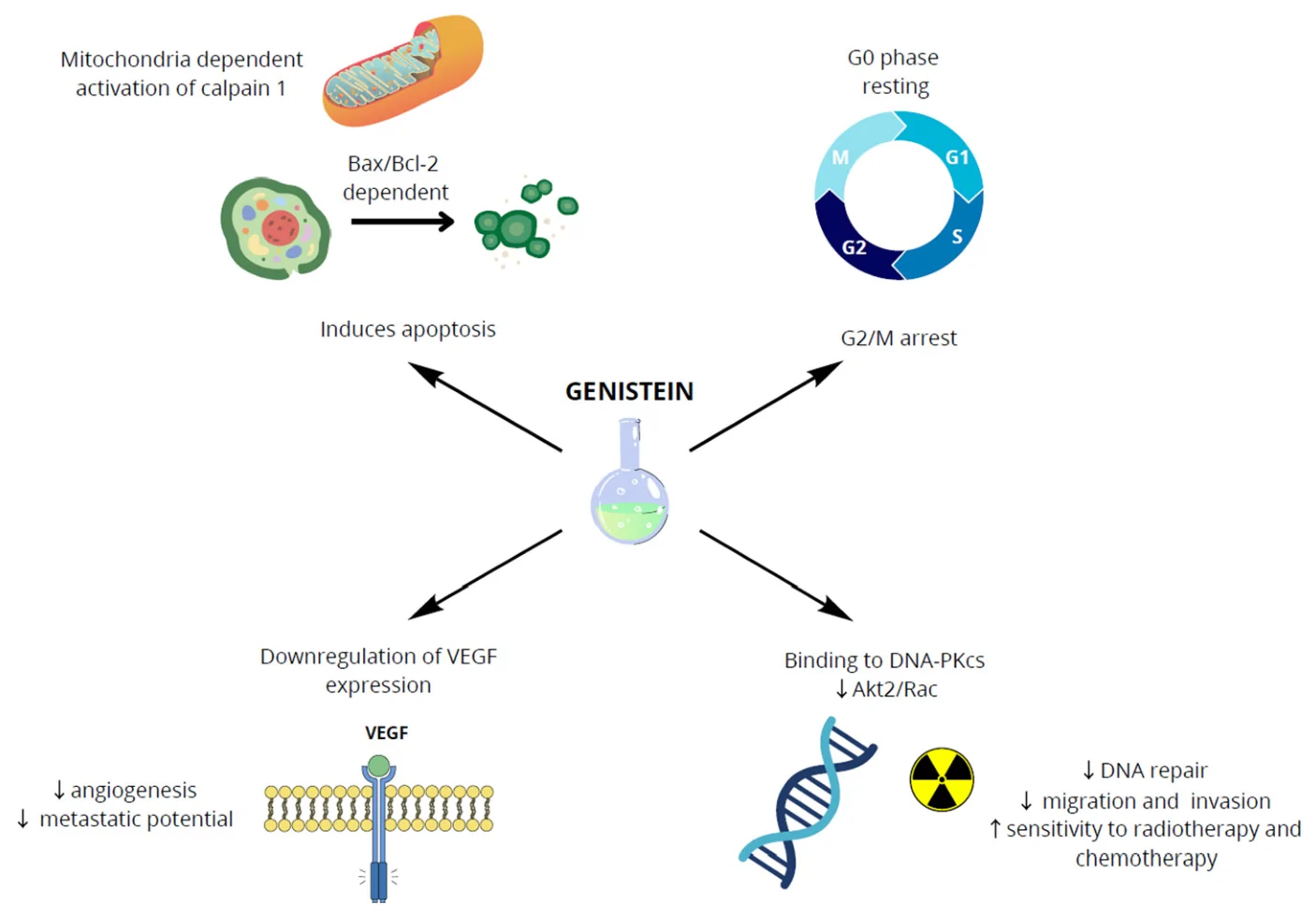
Summary of the anticancer mechanisms of action of genistein. Arrows are used schematically to indicate the relationships between genistein and the main mechanisms of action.

Nevertheless, higher local concentrations may be achievable using pharmaceutical formulations specifically designed to enhance tumor delivery, which remain an active area of ongoing research. A summary of the mechanisms of action of genistein is presented in Figure 10.

### 4.2. Genistein in Combination with Chemotherapeutics

Previously, the combined effects of genistein and temozolomide were evaluated using polymer-based nanoparticle delivery systems in glioblastoma U87MG cells, where a synergistic effect was observed [70]. However, nanoparticle-mediated delivery can significantly alter drug pharmacokinetics, cellular uptake, and intracellular distribution, which may contribute to the enhanced efficacy reported in that study. In contrast, our results obtained using free compounds revealed an antagonistic interaction in neural-derived tumor cell lines, highlighting the importance of drug formulation and experimental context in determining therapeutic outcomes.

Interesting results were observed in MCF-7 breast cancer cell lines. The combination of GEN and doxorubicin exhibited a synergistic effect on cancer cells, with GEN reducing the chemoresistance of these cells. This effect was likely associated with elevated intracellular accumulation of doxorubicin and the suppression of HER2 proto-oncogene expression by GEN [71]. Another study evaluated the effect of GEN in combination with doxorubicin-containing nanoparticles in the treatment of metastatic prostate cancer (RM-1). The combination of these two substances resulted in an increase in ROS production and intensification of oxidative damage in prostate cancer cells, leading to a significant reduction in tumor size and attenuation of prostate cancer metastases [72]. In a related study on prostate cancer, the impact of GEN treatment in combination with cabazitaxel on castration-resistant prostate cancer cells was examined. This combination enhanced the response to cabazitaxel treatment in cancer cells by increasing the expression of the proapoptotic protein Bax, activating apoptotic signals and enhancing the response to cabazitaxel treatment in cancer cells [73]. Moreover, there are documented reports on the beneficial interactions of GEN with other chemotherapeutic agents. The combination of GEN and cisplatin has been observed to enhance the apoptotic effect in cases of ovarian [74], cervical [75], breast, pancreatic and lung [76] cancer cell lines. GEN, in combination with 5-fluorouracil, demonstrated proapoptotic activity in colon [77,78] and pancreatic [79] cancer cells. In turn, the combination of GEN with gemcitabine has been shown significant outcomes in the in vitro treatment of pancreatic cancer [80].

A pilot study was also conducted in children receiving chemotherapy and/or radiotherapy to investigate the potential preventive effect of GEN toxicity. In children receiving GEN, compared to standard treatment, myelosuppression, mucositis, and infections were observed less frequently, and patients also reported less severe pain and diarrhoea [81].

The aforementioned studies suggest that GEN, when combined with standard treatment, has the potential to represent a promising research direction. Not only could it enhance the effect of chemotherapeutic agents on cancer cells, but it could also protect against the adverse effects of chemotherapy.

In the context of studies on TMZ combinations with naturally occurring substances, it is worth noting compounds other than GEN from the flavonoid group [82]. Interestingly, although both GEN and biochanin A belong to the isoflavone family, they do not necessarily exhibit identical biological activities. Biochanin A is an O-methylated derivative of genistein and differs in its physicochemical properties, cellular uptake and molecular targets. Previous studies demonstrated that biochanin A sensitizes glioblastoma cells to TMZ through modulation of AMPK/ULK1-dependent autophagy, p53 signaling, EGFR inhibition and cell cycle arrest [83,84]. In contrast, GEN has been shown to exert pronounced cytostatic effects, including G2/M cell cycle arrest and modulation of DNA repair pathways. Therefore, structurally related isoflavones may differentially influence TMZ-mediated cytotoxicity. These mechanistic differences could partially explain why biochanin A enhances TMZ activity whereas GEN exhibited antagonistic interactions in our neural-derived tumor models.

In the context of melanoma, a study evaluated the effects of doxorubicin in combination with GEN on SK-MEL-28 melanoma cells and healthy HaCaT keratinocytes. It was proved that GEN enhanced the sensitivity of SK-MEL-28 cells to doxorubicin, and that the combination of these two substances resulted in the death of cancer cells at lower concentrations compared to GEN and doxorubicin used separately. Furthermore, GEN was observed to reverse the cytotoxic effect of doxorubicin in the HaCaT line, indicating a chemopreventive effect on healthy keratinocytes. The molecular anticancer activity of this combination was primarily associated with promoting tubulin and F-actin constriction, increasing caspase-3, -7 and -9 activity, and increasing intracellular ROS levels [85]. The combination of GEN and TMZ has not been studied in melanomas to date. TMZ and doxorubicin are different drugs with different mechanisms of action, and their combination may lead to different interactions. Our experiment has shown, based on isobolographic analysis, that GEN+TMZ in melanoma was characterized by an additive interaction. The response of melanoma in this case is significantly different to the GEN+TMZ combination compared to the neural-derived tumors we studied, for which strong antagonism was observed. The difference between melanoma and neurological cancers with the GEN + TMZ combination most likely reflects tumor-type-specific biology, not just drug mechanism alone.

The divergent interaction between GEN and TMZ observed in melanoma versus neural-derived tumors may be associated with differences in tumor-specific biological characteristics. In melanoma, lower DNA repair efficiency (e.g., reduced MGMT activity or impaired mismatch repair) may allow TMZ-induced DNA damage to persist, leading to additive cytotoxic effects when combined with GEN [86]. Additionally, melanoma cells often exhibit high oxidative stress and dependence on pro-survival pathways such as MAPK and PI3K/AKT, which can be suppressed by GEN [59], thereby potentially modulating cellular responses to TMZ-induced damage. However, these proposed mechanisms were not directly investigated in the present study and should therefore be considered hypothetical. In the present study, molecular docking was used as an exploratory approach to investigate potential non-covalent interactions of GEN and intact TMZ with MGMT. Both compounds were predicted to occupy adjacent subregions within a broader region of MGMT surrounding the active-site Cys145, with Arg135 and Ser159 identified among the residues surrounding both ligands (Figure 9; Supplementary Table S2). However, neither GEN nor TMZ showed a direct contact with Cys145, and the docking results therefore do not demonstrate direct inhibition of MGMT. Furthermore, the use of the native, unalkylated form of Cys145 and intact TMZ as the docking ligand does not reproduce the covalent alkylation reaction or the conformational changes associated with MGMT after alkylation. Therefore, the predicted spatial proximity of the binding regions should not be interpreted as evidence that MGMT is directly responsible for the observed GEN–TMZ interaction or for the differences between tumor types. The docking results may serve as a hypothesis-generating basis for further investigation of potential molecular interactions, which would require validation using appropriate biochemical or biophysical approaches.

In contrast, the molecular basis of the antagonistic interaction observed between GEN and TMZ in neural-derived tumors remains unclear. One possible explanation is that GEN may alter cellular responses to DNA damage by modulating cell cycle progression and apoptosis-related signaling pathways. Previous studies have demonstrated that GEN is capable of inducing both cytostatic and cytotoxic effects depending on concentration, exposure time and tumor type. Therefore, it cannot be excluded that GEN-induced alterations in cell cycle dynamics or stress-response pathways influence TMZ-mediated cytotoxicity in neural-derived tumors. Alternatively, modulation of DNA repair pathways, oxidative stress responses or survival signaling networks may contribute to the observed antagonism. However, these mechanisms were not directly investigated in the present study and should be considered hypothetical. Differences in redox regulation and survival signaling between tumor types may alter how cells respond to combined treatment. Although the docking results indicate that GEN and TMZ may engage overlapping regions of MGMT, they do not provide an explanation for the tumor-specific difference in the pharmacological interaction observed in our study.

Overall, tumor-specific differences in cell cycle regulation, oxidative stress responses and signaling pathway dependence may contribute to the different responses observed in melanoma and neural-derived tumors following combined GEN and TMZ treatment. However, the molecular mechanisms underlying these interactions remain to be elucidated. These findings may have translational relevance, suggesting that genistein–chemotherapy interactions are highly tumor-dependent. In particular, the observed antagonism in neural-derived tumor models warrants further investigation of the potential interactions between genistein-containing supplements and temozolomide-based chemotherapy. Further in vivo and clinical studies are required to validate these observations and determine their clinical significance.

## 5. Conclusions

Effective drug combinations are constantly being sought, especially chemotherapeutic drugs with compounds of natural origin, the combination of which would produce a synergistic effect, which could reduce the dose of the drug used and limit its side effects. In our study, genistein demonstrated significant antiproliferative activity, with IC_50_ values ranging from approximately 40 to 110 μM in melanoma and 27 to 83 μM in neuroblastoma cell lines. The combination of GEN and TMZ produced additive interactions in melanoma cell lines, whereas a strong antagonistic interaction was observed in the investigated neural-derived tumor models. These findings suggest that the effects of the GEN+TMZ combination may be tumor-type-specific and should not be assumed to be universally beneficial. The present study has several important limitations. It was conducted entirely in vitro, used a single fixed GEN+TMZ ratio of 1:1, and included limited mechanistic validation. In addition, the mechanisms underlying the observed interactions were not directly investigated, and the effects were evaluated at a limited time point. Therefore, the potential therapeutic relevance of the GEN+TMZ combination should be interpreted cautiously. Further mechanistic and in vivo studies, together with pharmacokinetic and pharmacodynamic investigations, are necessary to validate these observations and assess their possible translational significance.

The antagonistic interaction observed in neural-derived tumor models also indicates that potential interactions between genistein-containing supplements and TMZ-based chemotherapy may warrant further investigation. However, the present in vitro findings do not support any conclusions regarding the clinical efficacy or safety of this combination.

## Figures and Tables

**Table 1:** The anti-viability effects of genistein and temozolomide on melanoma, neuroblastoma, and glioblastoma cell lines in the MTT assay.

	Cell Line/Drug	Genistein (µM)	Temozolomide (µM)	Temozolomide (µM) Previous Study [34]
neuroblastoma and glioblastoma	CHP-134	27.05 ± 1.78	262.59 ± 10.27	264.80 ± 14.84
	KELLY	60.01 ± 4.51	230.46 ± 29.08	227.75 ± 27.17
	U-87MG	83.86 ± 12.06	2390.18 ±509.07	2258.96 ± 621.93
	T98G	37.63 ± 9.87	1880.24 ± 363.53	1954.38 ± 339.37
melanoma	A375	40.80 ± 5.09	847.11 ± 122.27	
	SK-MEL28	110.09 ± 15.65	2054.33 ± 397.41	
	FM55P	86.23 ± 8.26	1219.25 ± 267.35	
	FM55M2	74.99 ± 6.48	940.15 ± 183.60	

The results are presented as IC_50_ values (means ± SEMs).

## Data Availability

The raw data supporting the conclusions of this article will be made available by the authors on request.

## Supplementary Materials

The following supporting information can be downloaded at https://www.mdpi.com/article/10.3390/cimb48101030/s1.

## Abbreviations

The following abbreviations are used in this manuscript:

IC_50_	Median inhibitory concentration
GEN	Genistein
TMZ	Temozolomide
