# Supplementary material for: Anticancer Effects of Genistein and Its Interaction with Temozolomide in Human Melanoma, Neuroblastoma and Glioblastoma Cells
Source: Curr Issues Mol Biol. 2026 Oct 4;48(10):1030. doi: 10.3390/cimb48101030 (PMC13703357; doi:10.3390/cimb48101030)
Supplement: Supplementary file 1 [file cimb-48-01030-s001.zip › cimb-4547818-supplementary.pdf]

current issues in molecular biology

Supplementary Materials

Anticancer effects of genistein and its interaction with temozolomide in human melanoma, neuroblastoma, and glioblastoma cells

Paula Wróblewska-Łuczka[1,*], Bartłomiej Kwiatkowski[1], Maciej Kwieciński[1], Agnieszka Góralczyk[1], Robert Domitrović[2] and Jarogniew J. Łuszczki[1]

[1] Department of Occupational Medicine, Medical University of Lublin, ul. Jaczewskiego 8b, 20-090 Lublin, Poland (paula.wroblewska-luczka@umlub.edu.pl (P.W.-Ł.); bartekchemlek@gmail.com (B.K.); m.kwiecinski.med@gmail.com (M.K.); agnieszka.goralczyk@umlub.edu.pl (A.G.); jarogniew.luszczki@umlub.edu.pl (J.J.L.))
[2] Department of Medical Chemistry, Biochemistry and Clinical Chemistry, Faculty of Medicine, University of Rijeka, B. Branchetta 20, 51-000 Rijeka, Croatia (robert.domitrovic@uniri.hr (R.D.))
* Correspondence: paula.wroblewska-luczka@umlub.edu.pl; Tel.: +48-81-448-6508

2. Materials and Methods

2.1. Cell Lines

Primary melanoma (FM55P) and metastatic malignant melanoma (FM55M2), two neuroblastoma (CHP-134 and KELLY) and normal keratinocytes HaCaT - human cell lines were cultured in RPMI-1640 Medium (Sigma-Aldrich, St. Louis, MO, USA). A375 cell line cultured in Dulbecco's Modified Eagle's Medium—high glucose (DMEM) (Sigma-Aldrich, St. Louis, MO, USA), SK-MEL28 and U-87MG cell lines were cultured in Eagle's minimal essential medium (EMEM), T98G and rat astrocytes were cultured in Dulbecco's Modified Eagle's Medium/Nutrient Mixture F-12 Ham (DMEM/F12, Sigma-Aldrich, St. Louis, MO, USA). All culture media used for human cell lines were supplemented with 10% Fetal Bovine Serum (FBS; Sigma-Aldrich, St. Louis, MO, USA) and 1% of penicillin/streptomycin (Sigma-Aldrich, St. Louis, MO, USA). The growth conditions of the neoplastic culture were described in details in the safety data sheet for each cell line (supported by ATCC and ECACC). Cultures were kept at 37 °C in a humidified atmosphere of 95% air and 5% CO2. The cells were maintained in the logarithmic growth phase by regular passage at 80% confluence.

2.2. Drugs

Genistein (Sigma-Aldrich, St. Louis, MO, USA) was dissolved in dimethyl sulfoxide (DMSO) to create a stock solution (200 mM). Temozolomide was dissolved in sterile water for injection as stock solutions in a concentration of 5 mg/mL. Before use, the drugs were dissolved to the respective concentrations in a culture medium, maintaining the concentration of DMSO at up to 0.1%. Because temozolomide and genistein were dissolved in different solvents (sterile water and DMSO), the untreated (control) cells were exposed to appropriate concentrations of the solvents in all experiments. For the two-drug mixtures, the control (untreated) cells were exposed to both solvents in the appropriate concentrations in all experiments. Temozolomide was used in combination with genistein in a fixed-ratio drug concentration of 1:1.

Academic Editor: Yoon-Jin Lee

Received: 21 August 2026
Revised: 27 September 2026
Accepted: 2 October 2026
Published: 4 October 2026

Copyright: © 2026 by the authors. Licensee MDPI, Basel, Switzerland. This article is an open access article distributed under the terms and conditions of the Creative Commons Attribution (CC BY) license.

2.3. Cell Viability Assessment - MTT assay

All the tested cell lines: A375, SK-MEL28, FM55P and FM55M2 (melanoma cells), CHP-134 and KELLY (human neuroblastoma cells), T98G and U-87MG (human glioblastoma cells), HaCaT (normal human keratinocytes) were placed on 96-well plates (Nunc, Roskilde, Denmark). The cell densities were: 2–3 × 10^4 cells/mL for melanoma cells, 1 × 10^5 cells/mL for CHP-134, KELLY, U-87MG cell lines and primary rat astrocytes, 1 × 10^4 cells/mL for T98G and HaCaT. The following day, the culture medium was removed and the cells were exposed to serial dilutions of temozolomide and genistein, both separately (at concentrations ranging from 2-200 μM for genistein and 100-2000 μM for temozolomide) in a fresh culture medium and in combination of temozolomide with genistein in a fixed-ratio drug concentration of 1:1. This ratio indicates that both drugs in mixture were combined at their IC_{50} values, based on the mass action law. Cells were exposed to temozolomide alone or to the temozolomide with genistein combination for 72 h without medium replacement.

Cell viability was evaluated after 72 h using the MTT colorimetric method which is based on the ability of viable cells to transform yellow, soluble tetrazolium salts [3-(4,5-dimethylthiazol-2-yl)-2,5-diphenyltetrazolium bromide, MTT] into purple, insoluble formazan, by cellular dehydrogenases. Cells were incubated for 3 h in the MTT solution (5 mg/mL, Sigma-Aldrich). Formazan crystals were solubilized overnight in sodium dodecyl sulfate (SDS) buffer (10% SDS in 0.01 N HCl) and the product was determined spectrophotometrically by measuring absorbance at 570 nm wavelength using microplate spectrophotometer (Ledetect 96, Labexim Products, Lengau, Austria). The 72-hour time point was selected to ensure sufficient exposure for detecting measurable effects on cell viability, and it corresponds to the average doubling time of all tested cell lines. Each experiment was performed in triplicate. Cell viability was normalized to control and determined by applying the following formula:

$$Cell\,Viability\,(\%) = \frac{Absorbance\ of\ treted\ cells}{Absorbance\ of\ control} \times 100$$

Control indicates cells subjected to solvents only (without genistein, temozolomide, or genistein + temozolomide treatment).

2.4. Cytotoxicity Assessment - LDH Assay

Optimized amounts of tested cancer cell lines and normal human keratinocytes (HaCaT) (1 × 10^4/mL), were placed on 96-well plates (Nunc, Roskilde, Denmark). The next day, cells were exposed to increasing concentrations of genistein in a fresh culture medium. The cytotoxicity was evaluated based on the measurement of cytoplasmic lactate dehydrogenase (LDH) activity released from damaged cells after 72 h exposure to genistein. LDH assay was performed according to the manufacturer's instruction (Cytotoxicity Detection KitPLUS LDH) (Roche, Mannheim, Germany). Absorbance was measured at two different wavelengths, one being the "measurement wavelength" (492 nm) and the other "reference wavelength" (690 nm) using a microplate spectrophotometer (Ledetect 96, Labexim, Lengau, Austria). Maximum LDH release (positive control) was achieved by the addition of Lysis buffer to untreated control cells. The average values of the culture medium background were subtracted from all values of experimental wells and the percentage of dead cells were calculated in relation to the maximum LDH release.

2.6. Isobolographic Analysis of Interactions

The interaction types between genistein and temozolomide were determined by isobolographic analysis method. First, the percent inhibition of cell viability per increas-

ing concentrations of genistein and temozolomide (when administrated alone in the cancer cell lines) was measured *in vitro* by the MTT assay. Then, the concentration-response effects for each investigated anti-cancer compound (i.e., genistein, temozolomide) were fitted with log-probit linear regression analysis as described by Litchfield and Wilcoxon. Subsequently, from the log-probit concentration-response lines, median inhibitory concentrations (IC_{50} values) together with their 95% confidence intervals, were calculated for GEN and TMZ.

Then, the concentration–response lines obtained for GEN and TMZ administered separately were tested for parallelism, and the median additive inhibitory concentrations (IC_{50add}) for the mixture of genistein with temozolomide were calculated. The experimentally-derived IC_{50mix} for the mixture of genistein with temozolomide (at the fixed-ratio of 1:1), inhibiting 50 % of cell viability in the tested cell lines, were determined in the MTT assay. This ratio was selected as the primary reference ratio to allow the effects of both compounds to be evaluated at equivalent relative concentrations. The IC_{50mix} value was then compared with the theoretically calculated IC_{50add} value. All experiments were performed in three independent biological experiments, with eight technical replicates per condition in each experiment. Thus, each concentration–response analysis was based on 24 measurements per cell line and treatment condition. The results were expressed as mean ± SEM.

The experimentally determined IC_{50mix} was compared with the theoretical IC_{50add} using an unpaired Student's t-test with Welch's correction. The interaction was interpreted as additive when the experimentally determined IC_{50mix} did not differ significantly from the theoretical IC_{50add}. A synergistic interaction was defined when IC_{50mix} was significantly lower than IC_{50add}, whereas an antagonistic interaction was defined when IC_{50mix} was significantly higher than IC_{50add}. To facilitate the interpretation of the isobolographic analysis, a representative schematic isobologram illustrating the theoretical additive area and the regions corresponding to synergistic and antagonistic interactions is presented in Figure S1. The schematic is intended to explain the principles of isobologram construction and interpretation and does not represent experimental data.

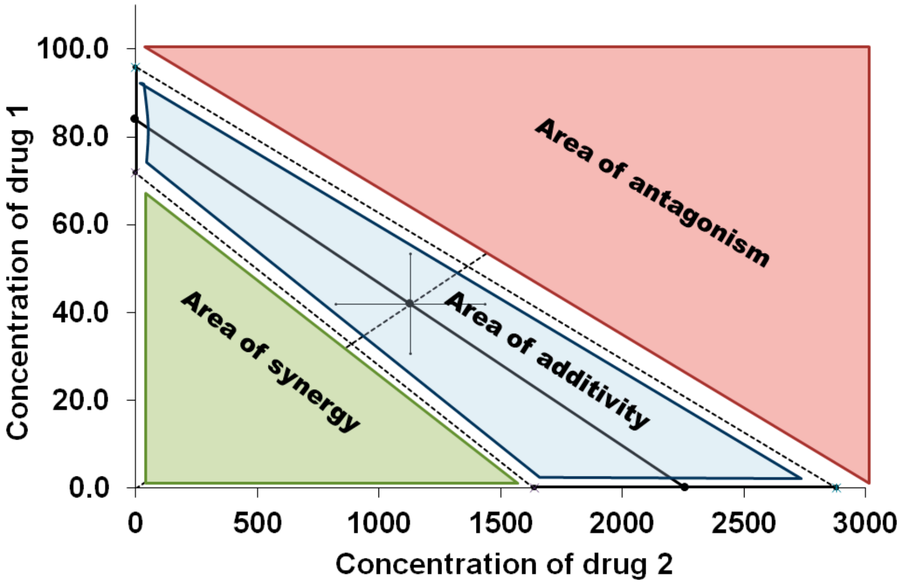

Figure S1. Schematic representation of isobolographic analysis. The solid diagonal line represents the theoretical additive line connecting the individual IC_{50} values of the two tested compounds on the x- and y-axes. The area below the additive line corresponds to synergistic interactions, whereas the area above the line represents antagonistic interactions. An experimental IC_{50}mix point located on the additive line indicates an additive interaction. The schematic is provided for illustrative purposes and does not represent experimental data.

https://doi.org/10.3390/cimb48101030

2.7. Statistical Analysis

Data from the MTT and LDH assays were first verified for their normal Gaussian distribution by means of the Shapiro-Wilk normality test and Kolmogorov-Smirnov test. Data showing a normal distribution were analyzed using one-way analysis of variance (ANOVA), followed by Dunnett's multiple-comparisons post hoc test, with each experimental group compared with the corresponding control group. The data are expressed as the mean ± standard error of the mean (SEM). (* $p < 0.05$, ** $p < 0.01$, *** $p < 0.001$, **** $p < 0.0001$). All experiments were performed in three independent biological experiments, with eight technical replicates for each experimental condition in each experiment. Thus, a total of 24 technical measurements were obtained for each condition and cell line. For each independent experiment, the eight technical replicates were first averaged to obtain one experiment-level mean. The overall mean was then calculated from the three independent experiment-level means, and the data are presented as mean ± SEM. Accordingly, the three independent experiments, rather than the individual technical measurements, were considered the biological replicates for statistical evaluation. The statistical analysis was conducted using GraphPad Prism 8.0 (San Diego, CA, USA). The IC_{50} and IC_{50mix} values for genistein and temozolomide, administered alone or in combinations at a fixed 1:1 ratio, were estimated separately for each of the three independent biological experiments using computer-assisted log-probit analysis based on the experiment-level mean concentration–response data obtained after averaging the eight technical replicates at each concentration. The corresponding IC_{50add} values were theoretically calculated from the experiment-level IC_{50} values according to the method described previously [42,43]. The experimentally derived IC_{50mix} values for the mixture of genistein and temozolomide were statistically compared with their respective theoretically additive IC_{50add} values by the use of unpaired Student's t-test with Welch's correction.

3. Results

3.1. Effect of genistein and temozolomide on cell viability in the MTT assay

https://doi.org/10.3390/cimb48101030

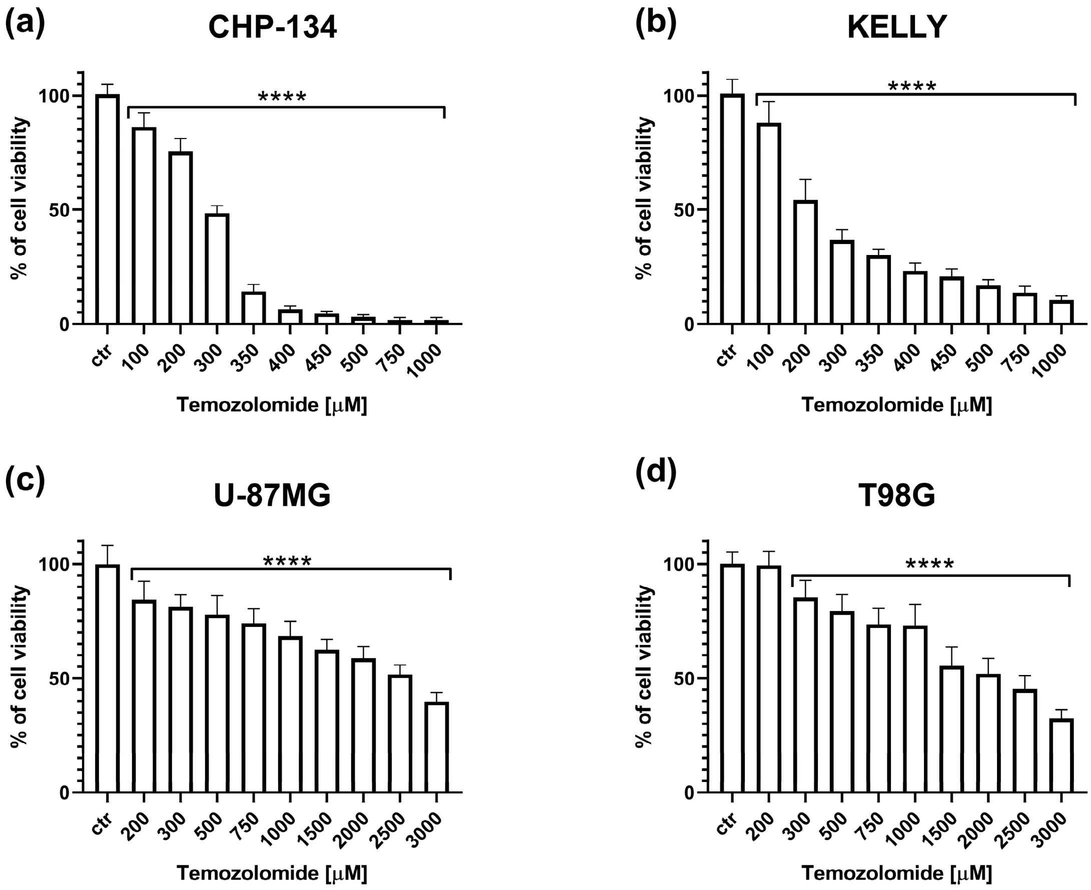

Figure S2. Concentration-dependent effects of temozolomide on the viability of neuroblastoma and glioblastoma cell lines, assessed by the MTT assay. Values are presented as mean ± SEM from three independent biological experiments, each comprising eight technical replicates per condition (24 measurements in total). **** $p < 0.0001$ vs. the control (ctr) group (one-way ANOVA followed by the Dunnett's post-hoc test).

3.3. Western Blotting Analysis

A prestained protein molecular weight marker (Perfect™ Tricolor Protein Ladder, EURx, Poland; E3210-01) was used as a size standard. The ladder contains proteins with molecular weights of 11, 17, 20, 25, 35, 48, 63, 75, 100, 135, 180, and 245 kDa.

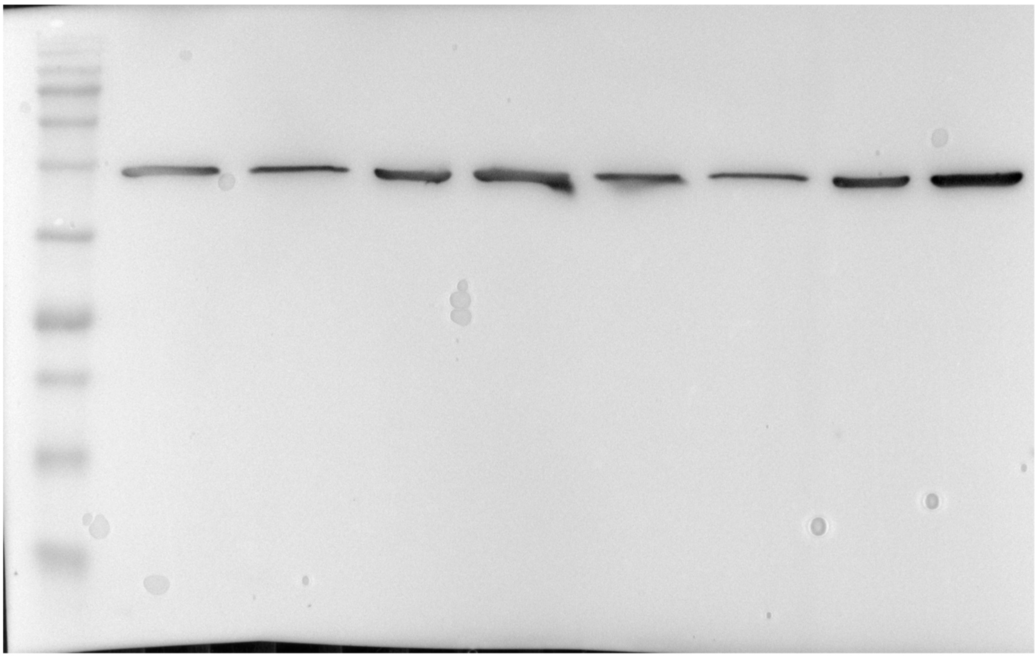

Figure S3. Representative Western blot analysis of β-actin expression (beta-Actin Antibody #4967, Cell Signaling, 45kDa) in melanoma (A375, FM55M2), glioblastoma (T98G), and neuroblastoma (CHP-134) cell lines treated with either culture medium (control, ctr) or genistein at IC_{50} concentration. β-actin was used as a loading control. Molecular weight markers (Perfect™ Tricolor Protein Ladder, EURx, Poland) are indicated. Blot signal (β-actin, 45kDa) is slightly below the Blot Marker band of 48 kDa.

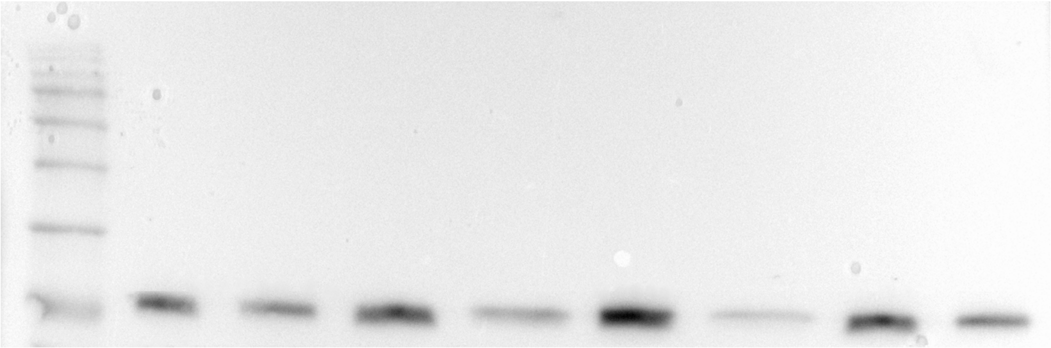

Figure S4. Representative Western blot analysis of Bcl-2 expression (Bcl-2 (124) Mouse Monoclonal Antibody #15071, Cell Signaling, 26 kDa) in melanoma (A375, FM55M2), glioblastoma (T98G), and neuroblastoma (CHP-134) cell lines treated with either culture medium (control, ctr) or genistein at IC_{50} concentration. Molecular weight markers (Perfect™ Tricolor Protein Ladder, EURx, Poland) are indicated. Blot signal (Bcl-2, 26 kDa) is slightly above the Blot Marker band of 25 kDa.

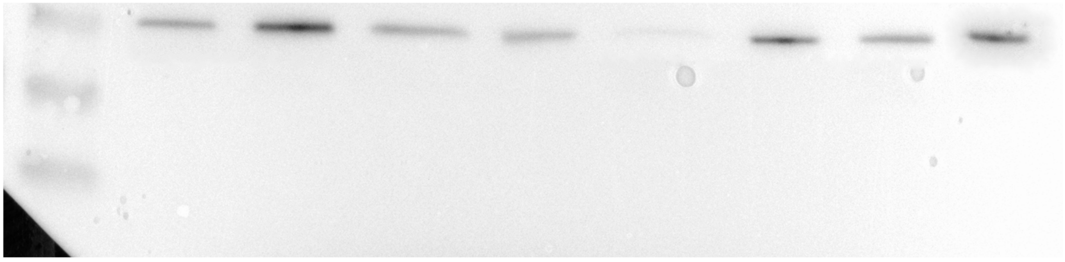

Figure S5. Representative Western blot analysis of Bax expression (Bax (E4U1V) Rabbit Monoclonal Antibody #41162, Cell Signaling, 20 kDa) in melanoma (A375, FM55M2), glioblastoma (T98G), and neuroblastoma (CHP-134) cell lines treated with either culture medium (control, ctr) or genistein at IC_{50} concentration. Molecular weight markers (Perfect™ Tricolor Protein Ladder, EURx, Poland) are indicated. Blot signal (Bax, 20 kDa) is at the height of the Blot Marker band of 20 kDa.

Table S1: Densitometric analysis of Bax, Bcl-2 and cl. caspase-3 protein expression in melanoma (A375, FM55M2), glioblastoma (T98G), and neuroblastoma (CHP-134) cell lines treated with culture medium (control, ctr) or genistein at IC_{50} concentration. Protein levels were quantified using FusionCapt Advance software and normalized to β-actin. All experiments were conducted independently at least three times (n=3), and the results are expressed as mean ± standard error of the mean (SEM).

Cell line	A375		FM55M2		T98G		CHP-134	
	ctr	IC_{50}	ctr	IC_{50}	ctr	IC_{50}	ctr	IC_{50}
Bax	4876 ± 18	11669 ± 23	4429 ± 11	3518 ± 19	1192 ± 12	4314 ± 14	5652 ± 14	9303 ± 22
Bcl-2	10467 ± 34	6771 ± 19	10879 ± 23	5163 ± 24	14448 ± 35	3077 ± 12	12464 ± 15	8678 ± 24
cl. caspase-3	629 ± 12	1409 ± 16	299 ± 11	568 ± 15	361 ± 11	970 ± 16	434 ± 12	4200 ± 17
β-actin	12646 ± 48	12188 ± 46	13737 ± 45	14177 ± 34	11814 ± 32	11449 ± 26	13833 ± 32	14047 ± 28

3.4. Isobolographic interactions between genistein and temozolomide in the MTT assay

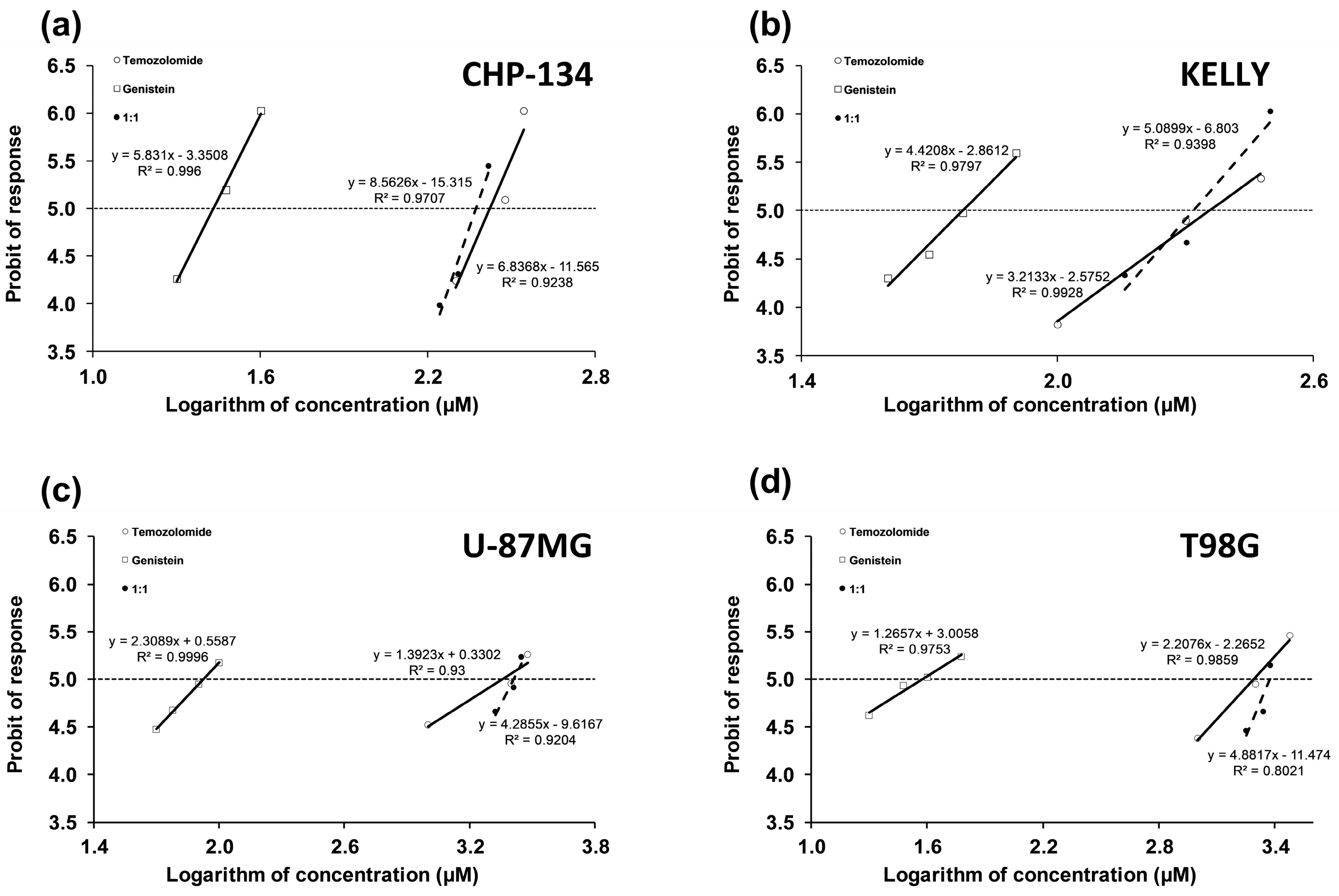

Figure S6. Log-probit concentration–effect lines for temozolomide and genistein, when administered alone and in combination in the fixed-ratio of 1:1, illustrating the anti-proliferative effects of the drugs in the various neuroblastoma, glioblastoma cell lines: CHP-134 (a), KELLY (b), U-87MG (c), T98G (d) measured in the MTT assay.

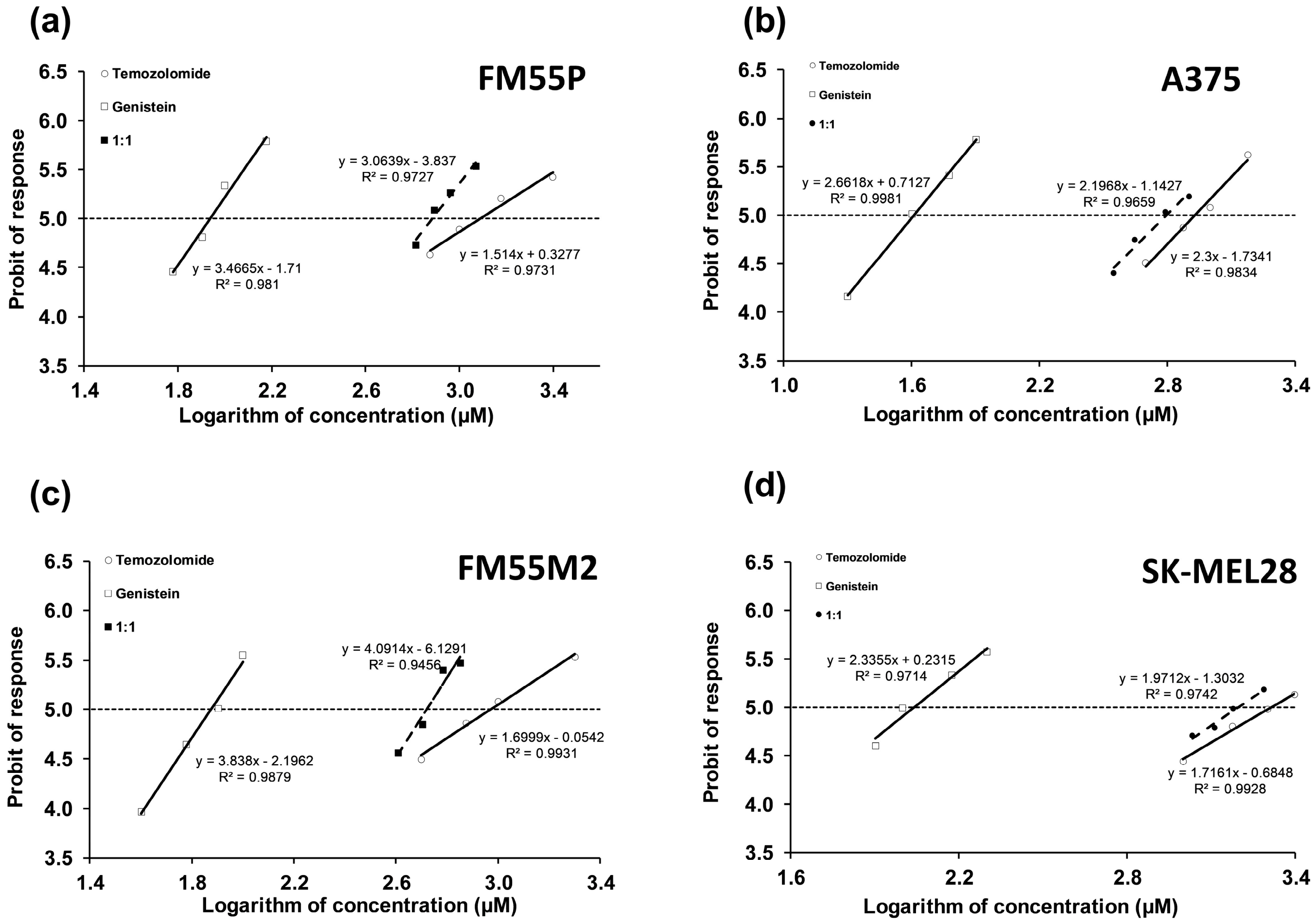

Figure S7. Log-probit concentration–effect lines for temozolomide and genistein, when administered alone and in combination in the fixed-ratio of 1:1, illustrating the anti-proliferative effects of the drugs in the various melanoma cell lines: FM55P (a), A375 (b), FM55M2 (c), SK-MEL28 (d) measured in the MTT assay.

https://doi.org/10.3390/cimb48101030

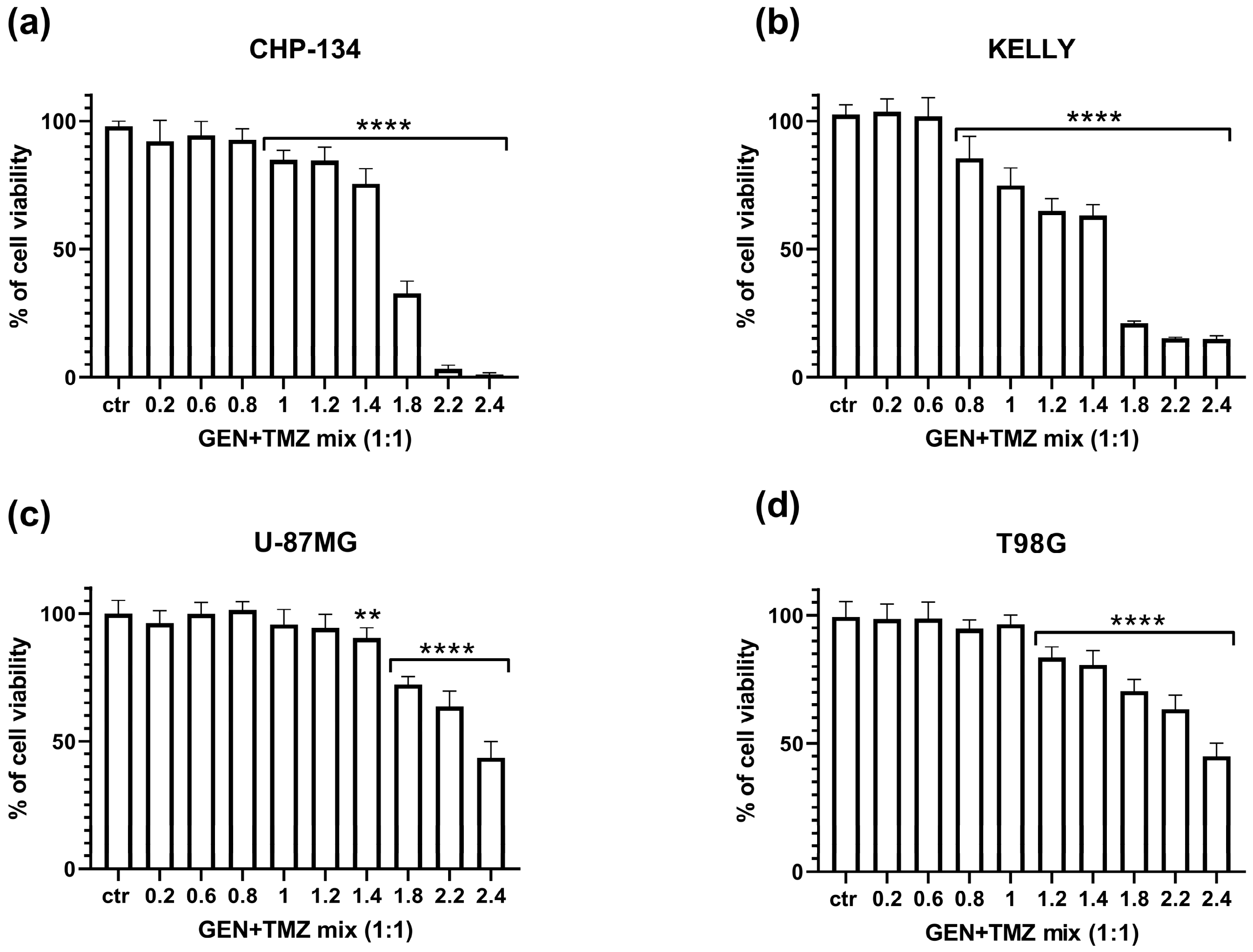

Figure S8. Concentration-dependent effects of the combination of genistein GEN with temozolomide TMZ (in a fixed ratio of 1:1) on the viability of neuroblastoma, glioblastoma cell lines (CHP-134 (a), KELLY (b), U-87MG (c), T98G (d)), assessed by the MTT assay. Values are expressed as mean ± SEM from three independent experiments, each performed with eight technical replicates (24 total measurements per cell line). ** $p < 0.01$, **** $p < 0.0001$ vs. the control (ctr) group (one-way ANOVA followed by the Dunnett's post-hoc test).

The values on axis X represent the multiplicity of calculated IC50 (1 means half IC50 of GEN + half IC50 of TMZ).

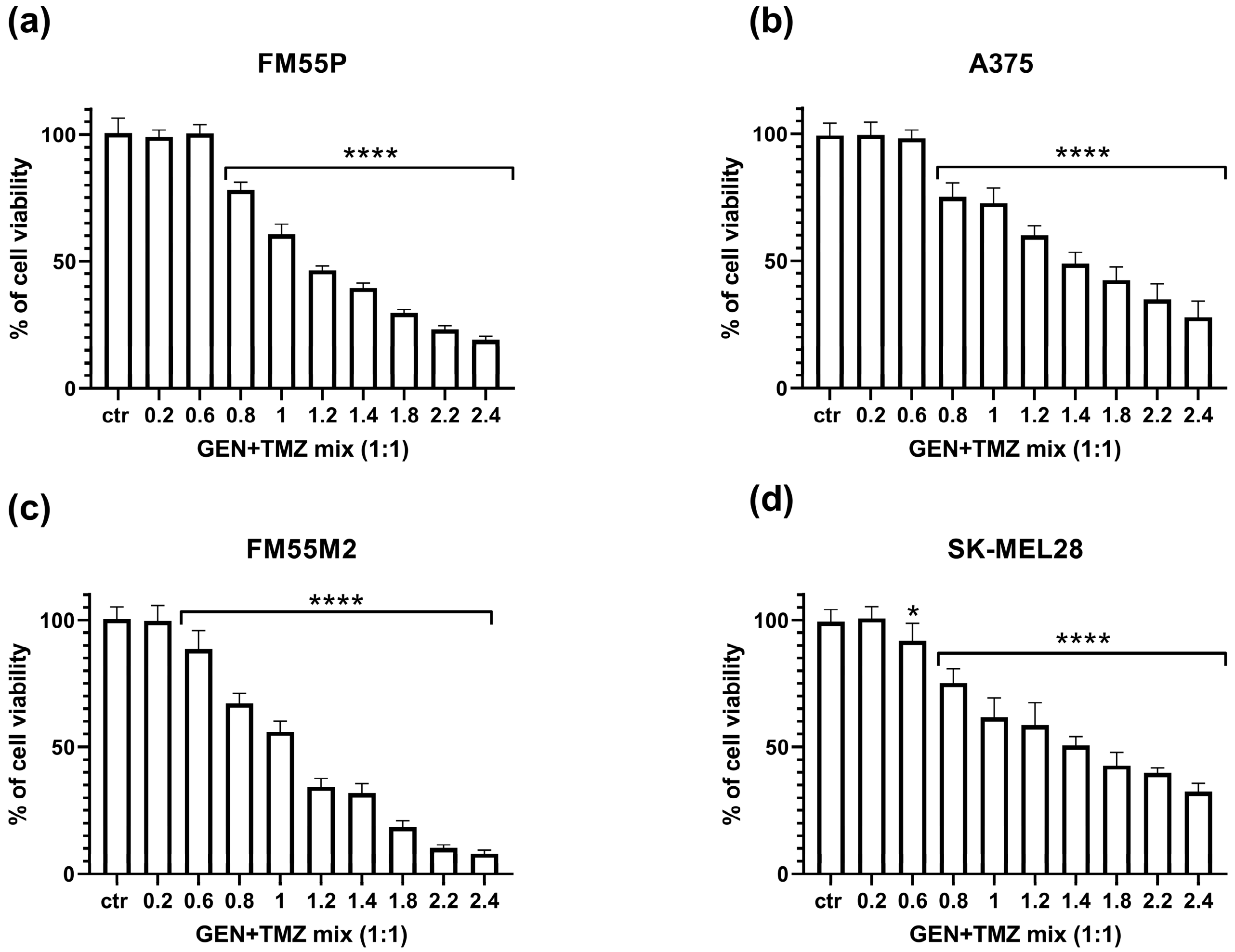

Figure S9. Concentration-dependent effects of the combination of genistein GEN with temozolomide TMZ (in a fixed ratio of 1:1) on the viability of melanoma cell lines (FM55P (a), A375 (b), FM55M2 (c), SK-MEL28 (d)), assessed by the MTT assay. Values are expressed as mean ± SEM from three independent experiments, each performed with eight technical replicates (24 total measurements per cell line). * $p < 0.05$, **** $p < 0.0001$ vs. the control (ctr) group (one-way ANOVA followed by the Dunnett's post-hoc test).

The values on axis X represent the multiplicity of calculated IC50 (1 means half IC50 of GEN + half IC50 of TMZ).

3.5. Molecular docking analysis of GEN and TMZ with MGMT

Table S2. Summary of molecular docking results for genistein (GEN) and temozolomide (TMZ) with human O6-alkylguanine-DNA alkyltransferase (MGMT). The table presents the top-ranked docking poses (Vina mode 1) obtained in two independent docking runs, predicted binding energies, minimum distances between the ligands and the sulfur atom of Cys145, potential hydrogen bonds identified using UCSF ChimeraX, and selected contacting MGMT residues. Both ligands showed reproducible positioning within the broader MGMT region surrounding Cys145, although neither ligand formed a direct contact with this residue. Arg135 and Ser159 were identified among

https://doi.org/10.3390/cimb48101030

the selected contacting residues for both ligands. No direct GEN–TMZ contacts were observed in the analyzed top-ranked poses.

Ligand	Docking run	Vina mode	Predicted binding energy (kcal/mol)	Minimum distance to Cys145-SG (Å)	Potential H-bonds	Selected contacting residues	Direct GEN–TMZ contacts
GEN	1	1	−5.794	4.94	1	Tyr114, Met134, Arg135, Asn157, Tyr158, Ser159	No
GEN	2	1	−5.787	5.09	0	Tyr114, Met134, Arg135, Asn157, Tyr158, Ser159	No
TMZ	1	1	−5.502	5.47	2	Leu33, Lys32, Arg135, Asn137, Pro138, Pro140, Ser159	No
TMZ	2	1	−5.503	5.47	2	Leu33, Lys32, Arg135, Asn137, Pro138, Pro140, Ser159	No

Disclaimer/Publisher's Note: The statements, opinions and data contained in all publications are solely those of the individual author(s) and contributor(s) and not of MDPI and/or the editor(s). MDPI and/or the editor(s) disclaim responsibility for any injury to people or property resulting from any ideas, methods, instructions or products referred to in the content.

https://doi.org/10.3390/cimb48101030
